# Supramolecular Engineering of a Homo[2]catenane Filler Enables Polymer Composites with Exceptional High-Temperature Capacitive Energy Storage

**DOI:** 10.3390/molecules31101691

**Published:** 2026-05-16

**Authors:** Qiao Su, Yan Sun, Jinfeng Li, Benteng Ma, Xiao Zhang, Haifeng Tian, Yuheng Ju, Saiwen Gao, Zhigang Liu, Tian Zhang, Lin Wu

**Affiliations:** 1 High Purity Chemistry Science and Technology Innovation Center of Jilin Province, Centre of Analysis and Measurement, Jilin University of Chemical Technology, 45 Chengde Street, Jilin 132022, China; 18893415189@163.com (Q.S.); 13331629907@163.com (B.M.); zx2561094342@163.com (X.Z.); 15512268629@163.com (H.T.); juyuheng_2002@163.com (Y.J.); 18103789927@163.com (S.G.); lzg@jlict.edu.cn (Z.L.); 2 Key Laboratory of Chemical Waste Resource Utilization of Jilin Province, School of Resources and Environment Engineering, Jilin University of Chemical Technology, 45 Chengde Street, Jilin 132022, China; sunyan@jluct.edu.cn; 3 Electronic Information School, Wuhan University, Wuhan 430072, China; 2025102120035@whu.edu.cn

**Keywords:** electrostatic interactions, electronic affinity, dielectric energy storage, high temperature, polyimide, homo[2]catenane, composites

## Abstract

The escalating demand for high-performance dielectric energy storage materials in pulse-power systems and portable electronics calls for polymer film capacitors with high discharged energy density and breakdown strength. Conventional polymers, however, suffer severe performance degradation under concurrent thermal and electrical stress, and existing reinforcement strategies—involving inorganic nanofillers or chemical crosslinking—often compromise flexibility, introduce interfacial defects, or involve complex processing. Herein, we demonstrate that incorporating a rigid mechanically interlocked molecule, specifically an octacationic homo[2]catenane, into a polyimide matrix yields robust, crosslink-like networks through strong [π∙∙∙π] electrostatic interaction between electron-rich aromatic units of polyimide and electron-deficient homo[2]catenane. This supramolecular network simultaneously enhances breakdown strength via densified chain packing and suppresses conduction loss by forming deep electron traps derived from the high electron affinity of homo[2]catenane. The optimized PI–HC^8+^ composite achieves a high discharged energy density of 7.86 J/cm^3^ with an efficiency > 80% and sustains stable performance over 10^5^ charge–discharge cycles at 150 °C. This research establishes mechanically interlocked molecules as a new class of functional fillers for high-performance polymer dielectrics, opening an unexplored avenue in the design of next-generation capacitive energy-storage materials.

## 1. Introduction

With the rapid advancement of renewable energy and advanced power electronics, highly efficient and reliable dielectric energy storage materials have become one of the core requirements for next-generation pulsed power systems and portable electronic devices [1,2,3,4,5,6]. Therefore, there is an urgent demand to develop high-performance dielectrics with stable energy storage capability over a broad temperature range [7,8]. Polymer dielectrics, owing to their low cost, high breakdown strength, low density, excellent mechanical flexibility, and unique self-healing capability, have been extensively explored for capacitor applications [9,10,11]. Currently, the most widely used dielectric polymer films are based on biaxially oriented polypropylene (BOPP). This material exhibits excellent processability and good self-healing properties, which typically operate at temperatures below 100 °C [12,13]. Furthermore, BOPP also suffers from a low discharged energy density (*U*_d_), which inevitably leads to a larger capacitor size in practical applications, failing to satisfy the demands of robust high requirements for dielectric energy storage capacitors [14,15]. Therefore, the development of polymer dielectrics that exhibit exceptional reliability and stability at elevated temperatures is of great significance.

A high glass transition temperature (*T*_g_) is a key criterion for polymeric dielectrics targeted at high-temperature applications, since polymer chains lose rigidity and free volume increases above the *T*_g_, leading to significant changes in dielectric constant (*ε*_r_) and loss factor (tan *δ*) [16,17]. Polyimide [PI], polyetherimide [PEI], fluorene polyester [FPE], polycarbonate [PC], and polyether ether ketone (PEEK) [18], owing to their high glass transition temperatures, are promising materials for high-temperature energy storage dielectrics [19,20]. Among them, PI is a linear dielectric, with a *T*_g_ that can reach up to 280 °C and with outstanding thermal stability [21]. The aromatic imide unit in PI’s molecular chain increases the polymer’s mechanical strength and heat resistance, while the ether unit provides mechanical flexibility and fluidity. Meanwhile, the stable *ε*_r_ and low tan *δ* make it an ideal choice for fabricating elevated temperature dielectric capacitors. Nevertheless, the breakdown strength (*E*_b_) and *U*_d_ of PI-based polymers decline abruptly under high temperatures and high electric fields, due to the increased conduction loss [22,23].

As the conduction loss is the main factor deteriorating the capacitive performance of dielectrics at elevated temperatures and high electric fields, suppressing the conduction loss of polymer dielectrics to improve their *U*_d_ and *E*_b_ is thus becoming a key strategy in order to overcome this challenge. To solve this issue, the construction of polymer-based composites with charge trapping and band gap modulation mechanisms have been proven to be an effective approach for suppressing conduction loss [24,25,26,27]. For example, Li and Wang et al. have shown that molecular semiconductors can act as carrier-trapping sites for capturing electrons and holes in the composites, thereby significantly reducing the leakage current density and improving *U*_d_ and *E*_b_ [18,28]. Another feasible strategy involves utilizing fillers to restrict polymer chain mobility via electrostatic attraction with PI chains [13,29,30]. Consequently, this effect promotes denser polymer chain packing and substantial mechanical reinforcement of the matrix, which in turn contributes to a significant enhancement in *E*_b_ and *U*_d_ [31,32].

Mechanically interlocked molecules (MIMs) are a class of molecules with mechanical bonds, which cannot be separated without breaking the participating covalent bonds [33,34,35,36]. Unlike traditional covalent or ionic bonds, these mechanical bonds are dynamic and non-covalent, often stabilized by supramolecular interactions such as hydrogen bonding, [π∙∙∙π] stacking or hydrophobic effects [37,38]. Catenanes are a special type of MIMs, which comprise two or more mechanically interlocked macrocycles, with potential functions from biomimetic systems to natural systems, and they have achieved notable progress in diverse research fields [39,40,41,42,43]. In comparison, catenanes act as a bridge between basic chemistry and applied materials engineering, yet the development of their unique 3D topological structures for applications in electronic devices—especially flexible ones—remains severely limited, primarily due to the challenges of precise structural regulation and efficient integration with polymer matrices. In 2013, Stoddart et al. reported on the synthesis by radical templating of a class of air- and water-stable 3D homo[2]catenane (HC^8+^) composed of two rigid and fixed CBPQT^4+^ rings [44]. The highly energetic octacationic 3D HC^8+^, which is capable of accepting up to eight electrons, can serve as a charge ‘reservoir’ [45]. Compared with traditional rigid inorganic fillers and chemically crosslinked polymers, the integration of HC^8+^ into polymer dielectric systems is expected to yield the following distinct advantages: (i) the structural tunability enables strong supramolecular interactions with the polymer matrix, allowing uniform molecular-level dispersion without obvious aggregation or phase separation and thus significantly enhancing interfacial compatibility; (ii) unrestricted polymer chain mobility, enabling reversible structural flexibility and good processability; and (iii) high-electron-affinity electron trapping can be achieved efficiently even at low doping concentrations. To the best of our knowledge, the 3D HC^8+^ is applied to the field of high-temperature energy storage for the first time.

**Figure 1:**
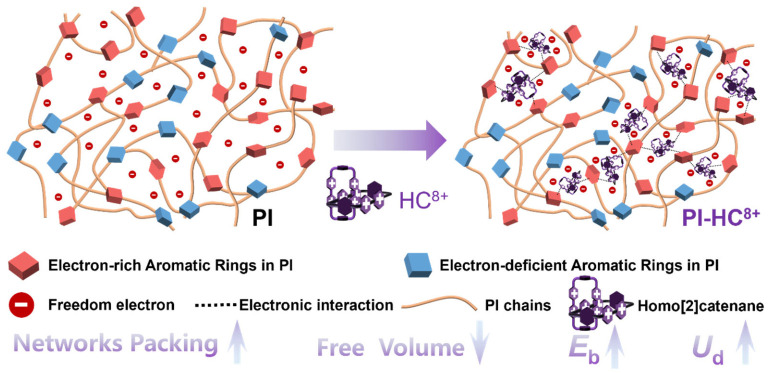
Schematic illustration of the polymer chain packing in pristine PI and PI-HC^8+^ composites.

Herein, we propose a novel filler design strategy based on homo[2]catenane, which simultaneously addresses the key challenges of high-temperature conduction loss and mechanical instability through a dual mechanism of electron trapping and electrostatic attraction. On one hand, the electron-withdrawing effect around the aromatic rings of 3D HC^8+^, via [π∙∙∙π] stacking interactions between the oppositely charged phenyl groups in PI and HC^8+^, can densify the polymer network (Figure 1). This assembly reduces the free volume of the polymer matrix, thereby enhancing the mechanical strength of PI and ultimately elevating its *E*_b_. On the other hand, the HC^8+^ has strong electron affinity, trapping electronic charge carriers to reduce the conduction loss. This approach opens a new avenue for MIMs in functional materials.

## 2. Results and Discussion

### 2.1. Density Functional Theory and Molecular Dynamics Simulation

**Figure 2:**
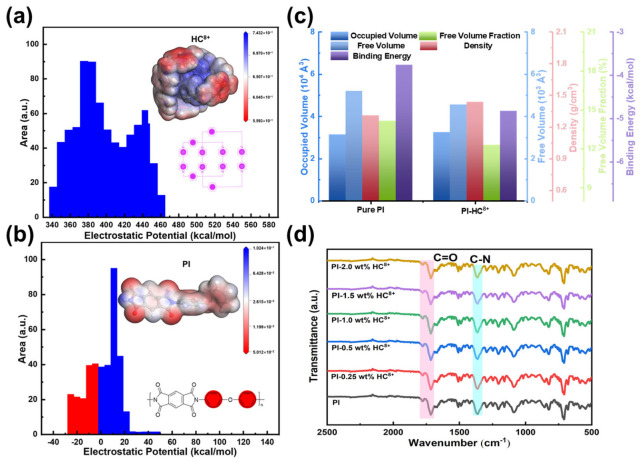
(**a**) Calculated electrostatic potential distribution and area in an HC^8+^ backbone. (**b**) Calculated electrostatic potential distribution of aromatic PIs in which the negatively charged phenyl groups are marked in red circles. (**c**) Calculated occupied volume, free volume, free volume fraction, density and binding energy. (**d**) FT-IR spectra of the raw PI and PI-HC^8+^ composites.

In order to explore the influence of HC^8+^ on the molecular chain structure of PI, we preliminarily calculated (Figure 2a,b) the distribution of electrostatic potential in HC^8+^ and PI using density functional theory (DFT). According to DFT calculations, some of the phenyl group combined with the imine group in the aromatic PI carries a negative potential (highlighted in red), while in the backbone of HC^8+^, it possesses a remarkable positive potential in the range of +340 to +460 kcal/mol on its surface, suggesting a possible electrostatic attraction between the HC^8+^ and electrons and an enhanced charge trapping capability of the HC^8+^ backbone.

To further quantify the electrostatic interaction strength between HC^8+^ and PI, we performed molecular dynamics (MD) simulations to calculate the binding energy of the systems. As shown in Figure 2c, the binding energy of pristine PI is −3.76 × 10^3^ kcal/mol, while that of the PI-HC^8+^ composite is −4.82 × 10^3^ kcal/mol, representing an approximately 28% increase. This substantial binding strength is attributed to the strong electrostatic attraction between the electron-deficient octacationic backbone of HC^8+^ and the electron-rich aromatic rings of PI. Notably, this binding energy far exceeds typical π-π stacking interactions (usually −5 to −15 kcal/mol), confirming the formation of a stable physical crosslinking network between HC^8+^ and PI.

To further understand the electrostatic interaction between them, we conducted (Figure S10, Supporting Information) MD simulations. The comparison of the calculated (Figure 2c) occupied volume, free volume, free volume ratio (FFV, free volume/sum of free volume and occupied volume), and density. According to the results, the initial free volume of pristine PI was about 5213 Å^3^ and the free volume fraction was 14.16%. After the addition of HC^8+^, these peaks decreased to nearly 4568 Å^3^ and 12.33%, respectively. Compared with the density of pure PI, nanocomposites exhibit higher densities. For example, the density of PI-HC^8+^ is 1.44 g/cm^3^ compared to that of PI, which is 1.31 g/cm^3^. The reduction in free volume and the increase in density in composites may be attributed to the electrostatic physical crosslinking effect of the addition of an appropriate amount of HC^8+^ on the polymer chains, which promotes the intermolecular forces and restricts the movement of the molecular chains. Therefore, it promotes a denser chain arrangement and significantly strengthens the polymers, suggesting a significantly enhanced breakdown strength and energy storage [13,32].

### 2.2. Fabrication and Characterization of Composite Films

**Figure 3:**
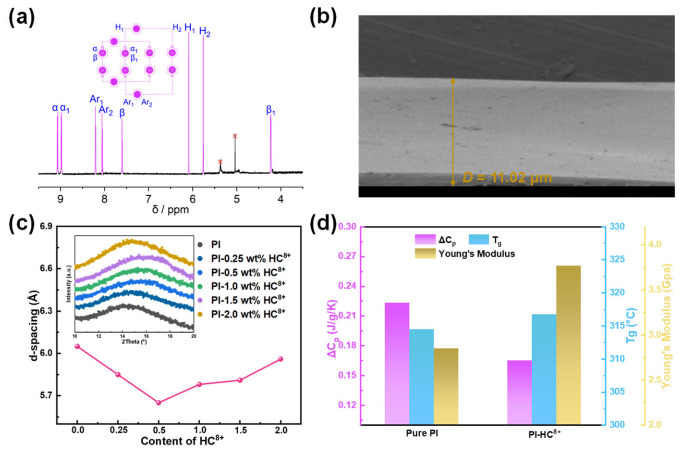
(**a**) The ^1^H NMR spectrum (600 MHz) of HC^8+^, recorded in CD_3_CN at 298 K, reveals resonances for the α_1_, β_1_, Ar_1_, and H_1_ protons associated with the outside BIPY^2+^ units in addition to resonances for the α_2_, β_2_, Ar_2_, and H_2_ protons associated with the inside BIPY^2+^ units. (**b**) Cross-section SEM of PI-0.5 wt% HC^8+^. (**c**) Interchain spacing calculated from XRD pattern. The inset is the XRD pattern of PI and different contents of PI-HC^8+^. (**d**) Δ*C*_p_, *T*_g_, and Young’s modulus from DSC curves and nano-indentation for pure PI and PI-0.5 wt% HC^8+^.

The compound of HC^8+^ was synthesized and characterized according to previously reported procedures (Figure S1, Supporting Information) [44]. Through treating one equivalent of *p*-xylylene dibromide with an excess of 4,4′-bipyridine, the XBPP is formed, followed by cyclization of XBPP with another equivalent of *p*-xylylene dibromide to give Blue Box, after purification and counterion exchange with NH_4_PF_6_ in H_2_O. One equivalent of Blue Box, XBPP, excess zinc powder, and 4,4′-bipyridine were added to MeCN. HC^8+^ was obtained after purification and counterion exchange with NH_4_PF_6_ in H_2_O and its structure was confirmed (Figure 3a and Figure S8, Supporting Information) by nuclear magnetic resonance (NMR) spectroscopy and high-resolution mass spectrometry (ESI-HRMS), respectively. Subsequently, PI-HC^8+^ composite films were prepared (Figure S9, Supporting Information) through typical solution casting techniques. To fabricate nanocomposite films with different filler contents, compounds of different mass fractions (0.25 wt%, 0.5 wt%, 1.0 wt%, 2.0 wt%) and 30 mg PI were first distributed ultrasonically into DMF for 15 min and then stirred for another 24 h at room temperature to ensure thorough mixing. Afterward, the solution was evenly spread on the glass plate by the pouring method. The film was dried at 70 °C for 12 h followed by 150 °C for 4 h to totally eliminate the solvent. Pure PI films without fillers were also prepared using the same procedure for comparison.

Fourier transform infrared spectroscopy (FT-IR) reveals the characteristic absorption peaks at 1780, 1717, and 1365 cm^−1^, representing the symmetric and asymmetric C=O stretching vibrations and the C–N stretching vibrations, respectively (Figure 2d). Moreover, the spectra of composites and PI are almost the same, which indicates that the chemical structure of the PI matrix is not affected by the doped filler. The film’s cross-section exhibits (Figure 3b and Figure S11, Supporting Information) a uniform and smooth surface without other defects from SEM micrographs at PI-0.5 wt% HC^8+^, indicating that the fillers have good compatibility with the polymer matrix. From the X-ray diffraction (XRD) result, there is only one broad diffuse peak for all samples at about 15 °. According to Bragg’s law, the interchain spacing of samples is calculated (Figure 3c). With the addition of fillers, the interchain spacing decreases from 6.04 Å for PI to 5.52 Å for PI-0.5 wt% HC^8+^ composites, revealing that the dense chain packing is realized. With the further increase in filler content, the interchain spacing of composites (>0.5 wt%) increases accordingly, probably a result of the excessive introduction of fillers, which imparts additional defects and accordingly augments the free volume within the material. Thermogravimetric analysis (TGA) results (Figure S13, Supporting Information) further confirm the thermal stability of the composites. The onset decomposition temperatures (*T*_d_) of pristine PI and the PI-0.5 wt% HC^8+^ composite are 535 °C and 520 °C under N_2_ atmosphere. Although the incorporation of HC^8+^ slightly reduces the decomposition temperature, it remains far above the operating temperature of 150 °C, demonstrating a sufficient thermal stability margin for the composites. The composite structure is further characterized (Figure 3d and Figure S12, Supporting Information) by *T*_g_ and the change in specific heat capacity (Δ*C*_p_) during the glass transition of PI and PI-0.5 wt% HC^8+^ composites, displaying *T*_g_ slightly increases from 314.5 °C for PI to 316.7 °C for PI-0.5 wt% HC^8+^, indicating the structural stability of composites at high temperature. More importantly, Δ*C*_p_ is substantially reduced from 0.223 J/g/K for PI to 0.165 J/g/K for PI-0.5 wt% HC^8+^, which means the electrostatic attraction between HC^8+^ and PI matrix straightens the PI chains compared to the naturally self-entangled state [22]. Young’s modulus of composites is significantly improved (Figure 3d and Figure S14, Supporting Information) from 2.81 GPa for PI to 3.67 GPa for PI-0.5 wt% HC^8+^. The improvement of Young’s modulus of the films benefits the resistance to electrical breakdown of dielectrics according to the electromechanical breakdown mechanism, which helps in restraining the mechanical collapse of dielectrics at high temperatures and high electric fields. To summarize, the construction of an electrostatic physical crosslinking network densifies polymer chain packing efficiently, thereby contributing to improved electrostatic energy storage properties.

### 2.3. Dielectric Properties

**Figure 4:**
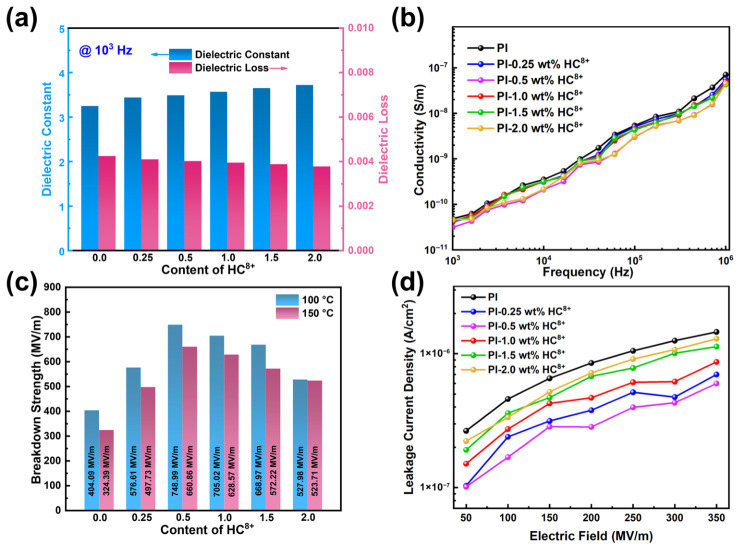
(**a**) The comparison of *ε*_r_ and tan *δ* for PI and PI-HC^8+^ composites. (**b**) The conductivity of PI and PI-HC^8+^ composites at different frequencies. (**c**) Weibull distribution analysis of the breakdown strength for PI and PI-HC^8+^ composites at 100 °C and 150 °C. (**d**) Leakage current density versus electric field for PI and PI-HC^8+^ composites at 150 °C.

Dielectric properties, including *ε*_r_ and dielectric loss (tan *δ*), are crucial for enhancing the energy storage capabilities of film capacitors. To investigate the dielectric properties of composites, we examined *ε*_r_ and tan *δ* across various frequencies and temperatures. As seen, the variations in *ε*_r_ and tan *δ* of all polymers are within the frequency range from 10^2^ Hz to 10^6^ Hz at room temperature. With the increase in frequency, the *ε*_r_ of composites shows a gradually decreasing trend, while the tan *δ* shows a corresponding gradually increasing trend (Figure S15, Supporting Information). This behavior may be attributed to the dielectric relaxation phenomenon [46,47,48]. As depicted in Figure 4a, the *ε*_r_ of the composites slightly increases with the rise in HC^8+^ contents at a constant frequency. This phenomenon may be attributed to the interface polarization from HC^8+,^ which generates the improvement in *ε*_r_, and the contribution formed from the HC^8+^ at the PI matrix interface. Simultaneously, the tan *δ* of PI-based composites is lower than that of pristine PI, which remains below 0.005 between 10^2^–10^6^ Hz frequency range owing to the outstanding interfacial compatibility of the PI matrix. By analyzing the impact of incorporating HC^8+^ on the thermal stability of *ε*_r_ and tan *δ* in the composites, we examined the dielectric characteristics of polymer films over a temperature range of room temperature to 150 °C at 10^3^ Hz (Figure S16, Supporting Information). The results indicate that with increasing temperature, the *ε*_r_ and tan *δ* of composites remain stable, suggesting excellent thermal stability for the PI-HC^8+^ composites. Although the tan δ increases with the raised temperature, they are still kept at a low level (<0.01).

The dielectric application scenario requires that the dielectrics need good insulation properties, which are usually judged by Weibull breakdown strength [49,50]. To investigate the impact of HC^8+^ on the *E*_b_ of PI thin film dielectric materials, the two-parameter Weibull distribution was utilized. Figures S17 and S18 (Supporting Information) display the Weibull distribution of pure PI and PI-HC^8+^ composites with varying filler contents at 100 °C and 150 °C, respectively. All *E*_b_ values are lower at 150 °C compared to those at 100 °C in the same contents due to increased leakage current within the composites under an external electric field. PI-0.5 wt% HC^8+^ at 100 °C achieves a high *E*_b_ value of 748.99 MV/m, which is 1.85 times greater than that for pure PI (404.09 MV/m). Even at 150 °C, PI-0.5 wt% HC^8+^ composite still exhibits an *E*_b_ of 660.86 MV/m, which is two times higher than that of PI (324.39 MV/m). Furthermore, the shape parameter (*β*) of determined by Weibull statistical analysis also increases substantially from 9.56 to 18.4 for PI-0.5 wt% HC^8+^ at 150 °C, demonstrating high homogeneity and superior reliability of the composites. Therefore, enhancement of the *E*_b_ in the PI-based composite film may be attributed to the unique electrostatic distribution of HC^8+^. Through intermolecular electrostatic interactions, the aggregation structure of PI becomes denser, while the in-plane orientation of the polymer chains is weakened. This disruption of the conjugation relationship between aromatic rings ultimately inhibits charge conduction [31]. The inclusion of additional filler content leads to agglomeration among the fillers, which facilitates the formation of conductive pathways and consequently reduces the *E*_b_ of the material.

Additionally, the improved *E*_b_ verifies the reinforcing effect of the physical crosslinking networks in PI-HC^8+^ composites, consistent with the above Young’s modulus results. Additionally, the enhanced *E*_b_ values of PI-HC^8+^ composites are further corroborated by the reduced leakage current observed at high temperatures (Figure 4d). Specifically, at 350 MV/m and 150 °C, the leakage current density (*J*) drops from 1.45 × 10^−6^ A/cm^2^ for pure PI to 5.99 × 10^−7^ A/cm^2^ for the PI-0.5 wt% HC^8+^ composite, manifesting a marked decline in conduction loss and improved efficiency (*η*). This significant leakage current reduction is attributed to the suppressed electronic charge transport in the composite: the backbone of HC^8+^ possesses inherently higher electron affinity than the PI matrix, forming carrier traps that efficiently capture electronic carriers. Meanwhile, the electron-deficient backbone of HC^8+^ forms strong electrostatic interactions with PI via oppositely charged phenyl groups, which densify the network of polymer chain and thus inhibit charge conduction. We then study the charge carrier trapping characteristic of PI and PI-HC composites by thermally stimulated depolarization current (TSDC) curves, as shown in Figure S19 (Supporting Information). The TSDC results reveal that, compared with pristine PI, the depolarization current peak of the PI-0.5 wt% HC^8+^ composite shifts to a higher temperature with a significantly increased intensity, indicating that the introduction of HC^8+^ constructs deeper and denser electron traps in the PI matrix. This trend is highly consistent with the reduced leakage current observed in Figure 4d, further confirming that the deep traps introduced by HC^8+^ effectively suppress charge carrier transport and conduction loss.

### 2.4. Energy Storage Capability

**Figure 5:**
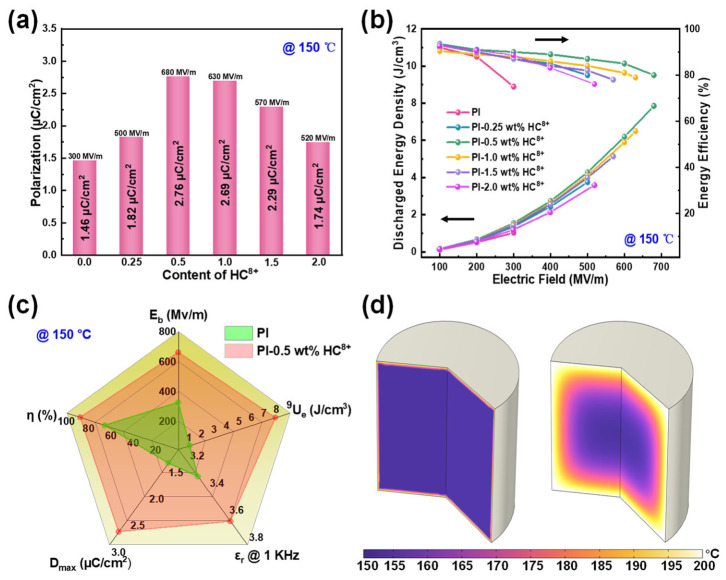
(**a**) Comparison of the maximum polarization in pure PI versus PI-HC^8+^ composites at 150 °C. (**b**) Energy storage performance of pristine PI and PI-HC^8+^ composites at 150 °C and 10^3^ Hz. (**c**) The radar plot. (**d**) Simulated rise in temperature of capacitors constructed from pristine PI and PI-0.5 wt% HC^8+^ composite under sustained operation at 300 MV/m and 150 °C.

By analyzing the electric-displacement–electric-field (*D*-*E*) loops (Figures S21 and S23, Supporting Information), we assessed the energy storage performance of PI and PI-HC^8+^ composites with various ratios at different temperatures. It is obvious (Figure 5a) that the maximal *E* and *D*_max_ of the composite films firstly rise and then fall as the HC^8+^ content increases, yet they overall exceed those of the original PI. For instance, the PI-0.5 wt% HC^8+^ composite achieves *E*_max_ of 680 MV/m at 150 °C, which is 2.26 times higher than that of pristine PI (300 MV/m), while its *D*_max_ at 680 MV/m (2.76 µC/cm^2^) is 1.89 times greater than that of pure PI (1.46 µC/cm^2^).

Integral calculations were carried out (Figure S18, Supporting Information) on the *D*-*E* loops of PI-HC^8+^ composites to derive the *U*_d_ and *η*. Thanks to the synergistic enhancement of *ε*_r_ and *E*_b_, the energy storage performance of composites is superior to that of pure PI. Obviously, at 100 °C, the PI-0.5 wt% HC^8+^ composite (Figure S22, Supporting Information) exhibits an *U*_d_ of 8.16 J/cm^3^ with *η* over 85%, which is 3.84 times higher than that of the raw PI (2.12 J/cm^3^). With increasing temperature up to 150 °C, pristine PI experiences a significant reduction in *E*_b_ and *U*_d_ as a result of elevated leakage current. Conversely, HC^8+^ fillers effectively enhance the thermal stability of PI, such that PI-HC^8+^ composites maintain superior energy storage performance. The PI-0.5 wt% HC^8+^ composite achieves a maximum *U*_d_ of 7.86 J/cm^3^ at 680 MV/m and 150 °C, which is 7.48 times greater than that of pristine PI (1.02 J/cm^3^ at 300 MV/m). Simultaneously, *η* is also improved: the PI-0.5 wt% HC^8+^ composite not only exhibits elevated *η* values but also maintains a high efficiency level exceeding 80% at 150 °C even under an electric field of up to 680 MV/m. In addition, a direct visual comparison (Figure 5b) of the maximum *U*_d_ and *η* values between raw PI and PI-HC^8+^ composites with different filler loadings indicates that all PI-HC^8+^ composites show a remarkable enhancement in their *U*_d_ and *η* relative to pristine PI. Furthermore, the unique advantage of the low content of HC^8+^ enables the composite to achieve higher energy density compared to pure PI at high temperatures. Correspondingly, Figure 5c compiles the key dielectric energy storage performance metrics—*U*_d_, *η*, *E*_b_, *ε*_r_ and *D*_max_—for both pristine PI and the PI-0.5 wt% HC^8+^ composite at 150 °C. In comparison with pure PI, the composite displays hysteresis loops with a larger enclosed area, which testifies to the achievement of well-balanced, comprehensive dielectric energy storage performance for the modified composite. These findings confirm that the incorporation of HC^8+^ effectively enhances the dielectric energy storage performance of PI. This performance enhancement can be attributed to the reduced leakage current and lowered conduction loss, which stem from the synergistic effect of physical crosslinking between HC^8+^ and PI matrix, as well as the introduction of charge traps.

**Figure 6:**
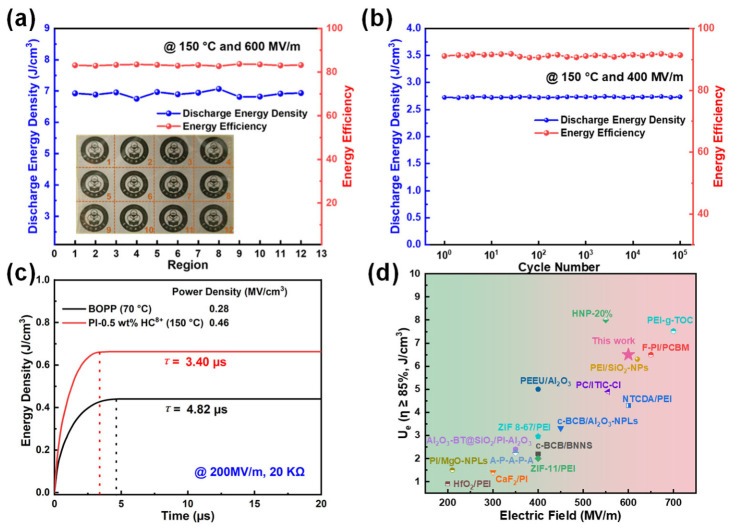
(**a**) Energy storage performance of PI-0.5 wt% HC^8+^ at different regions at 600 MV/m and 150 °C. (**b**) Cyclic performance of PI-0.5 wt% HC^8+^ at 400 MV/m and 150 °C. (**c**) Fast discharge testing of PI-0.5 wt% HC^8+^ (200 °C) and BOPP (70 °C) at 200 MV/m and 20 kΩ. (**d**) Comparison of the *U*_d_ above 85% efficiency of PI-0.5 wt% HC^8+^ and recently published work at 150 °C.

To ensure uniform energy storage performance and good film quality for the composite, a large 10 × 10 cm^2^ film was fabricated (Figure 6a) and divided into twelve regions to characterize its energy storage performance. The *U*_d_ and *η* of the different regions exhibited high consistency at 600 MV/m and 150 °C, which demonstrates excellent uniformity of the film. We also simulated the temperature rise of capacitors at 300 MV/m and 150 °C via COMSOL 6.4 Multiphysics, with pristine PI and PI-0.5 wt% HC^8+^ composites serving as dielectric films. The capacitor with raw PI exhibits (Figure 5d) a more obvious temperature increase than that with PI-0.5 wt% HC^8+^ composites as the dielectric layer. This mitigated temperature rise is mainly attributed to the suppressed conduction and energy losses, which stem from the introduction of PI-HC^8+^ particles into the PI matrix.

### 2.5. Reliability and Stability Performance

Beyond pursuing high energy storage performance, reliability and stability are also essential considerations for practical applications. To validate the cyclic stability of samples under extreme conditions, cyclic charge–discharge tests were conducted (Figure 6b) on pristine PI and PI-0.5 wt% HC^8+^ composite at 400 MV/m and 150 °C, revealing that the *U*_d_ and *η* of the composite nearly remained stable after 10^5^ cycles. The fast discharge performance of PI-0.5 wt% HC^8+^ was also evaluated (Figure 6c). Its discharge time was 3.40 μs at 150 °C, much shorter than that of biaxially oriented polypropylene (BOPP) measured at 70 °C (4.82 μs). In addition, the power density of PI-0.5 wt% HC^8+^ reached 0.46 MW/cm^3^, over twice that of BOPP (0.28 MW/cm^3^), which confirms the significantly improved discharge performance of PI-HC^8+^ composites. Finally, the energy storage performance of PI-HC^8+^ composites is benchmarked against other recently reported dielectric materials for high-temperature applications at 150 °C. Notably, the PI-0.5 wt% HC^8+^ composite delivers (Figure 6d) exceptional energy storage performance at this temperature [19,47,51,52,53,54,55,56,57]. For instance, PEI-Ni@MOFs (6.37 J/cm^3^ at MV/m), PEI-AIN (4.79 J/cm^3^ at 520 MV/m) and PEI-TCNQ (7.84 J/cm^3^ at 670 MV/m) all exhibit lower performance than the PI-0.5 wt% HC^8+^ composite, which attains 7.86 J/cm^3^ at 680 MV/m. Furthermore, compared to the BaMoO_4_/Zn-BaMoO_4_ hybrid supercapacitor reported by Dorji et al. [58], our PI-0.5 wt% HC^8+^ achieves a comparable energy density while demonstrating superior performance. Collectively, these results show that the nanocomposites produced here have great prospects in high-temperature dielectric energy storage applications.

## 3. Materials and Methods

### 3.1. Film Preparation

The composite films were prepared by the solution casting method. First, PI was dissolved in DMF at a concentration of 20 mg/mL. Compounds with different doping ratios were then added to the solution, and the mixture was stirred for 24 h. The solution was then drop-cast onto pre-cleaned glass slides and dried at 70 °C for 12 h to remove the solvent and then heated to 150 °C for 2 h. The films were further dried in vacuum at 50 °C for 5 h to completely remove the residual solvent. Finally, the films were peeled off of the glass slides.

### 3.2. Structural Characterizations

All solvents and reagents were commercially available and used without further purification unless otherwise noted. Reactions were monitored by thin-layer chromatography (TLC) using precoated silica gel plates. Silica gel (200–300 mesh) was used as the stationary phase of column chromatography during purification. Solution nuclear magnetic resonance (NMR) spectra were recorded on Bruker AVANCE NEO 600 MHz (Bruker, Billerica, MA, USA), equipped with 5 mm inverse quadruple resonance QCI-F CryoProbe. Working frequencies were 600 MHz for ^1^H. Chemical shifts are reported in parts per million (ppm) relative to the signals corresponding to the residual non-deuterated solvents (CD_3_CN: *δ*_H_ = 1.94 ppm; CDCl_3_: *δ*_H_ = 7.26 ppm). The following abbreviations (or combinations thereof) were used to explain the multiplicities: s = singlet, d = doublet, t = triplet, q = quartet, dd = doublet of doublets, m = multiplet, and br = broad. High-resolution mass spectra (HRMS) were analyzed with ultra-performance liquid chromatography coupled with a Waters Synapt-G2-Si Electrospray Ionization Time-of-Flight (ESI-TOF) mass spectrometer (Waters, Milford, MA, USA). The analysis was performed in positive ion mode with an m/z range from 150 to 1200 Da. Fourier transform infrared (FT-IR) spectra were conducted on a Thermo Fisher Nicolet iS50 spectrometer (Thermo Fisher Scientific, Waltham, MA, USA) in attenuated total reflectance (ATR) mode over the range of 4000–400 cm^−1^. XRD analysis was performed using an X-ray diffraction instrument (XRD, Bruker D8, Bruker, Billerica, MA, USA). Thermal stability tests were conducted employing a thermogravimetric analyzer (TG, Discovery SDT650, TA Instruments, New Castle, DE, USA). The microstructure and elemental distribution of the obtained material were analyzed via field emission scanning electron microscopy (FESEM, Jeol JSM-7610FPlus, JEOL, Tokyo, Japan).

## 4. Conclusions

In summary, we have designed and prepared polyimide-based organic composites incorporating homo[2]catenane by means of a straightforward solution-casting method. This strategy combines the benefits of a high-electron-affinity filler with [π∙∙∙π] electrostatic interaction between the filler and the matrix. The introduction of an octacationic homo[2]catenane with high electron affinity successfully constructs electron traps that suppress electronic conduction. Concurrently, the electrostatic interaction between the strongly electron-deficient backbone of HC8+ and the electron-rich aromatic moieties in the polyimide matrix enhances mechanical strength. Benefiting from this synergistic effect, the composites exhibit improved dielectric energy-storage performance compared to pristine polyimide. Specifically, the composite with 0.5 wt% HC^8+^ achieves outstanding high-temperature energy densities of approximately 8.47 J/cm^3^ at 100 °C and 7.86 J/cm^3^ at 150 °C. This research not only offers a novel approach to preparing dielectric polymers with high energy density and efficiency under harsh conditions employing mechanically interlocked molecules as fillers but also pioneers a new direction for the application of mechanically interlocked molecular architectures.

## Data Availability

Any additional data related to this paper are available upon sending a request to the authors.

## Supplementary Materials

The following supporting information can be downloaded at: https://www.mdpi.com/article/10.3390/molecules31101691/s1. Experimental details, as well as figures related to the experiments discussed in the manuscript.

## Abbreviations

The following abbreviations are used in this manuscript:

PI	Polyimide
PEI	Polyetherimide
FPE	Fluorene Polyester
PC	Polycarbonate
PEEK	Polyether Ether Ketone
MIMs	Mechanically Interlocked Molecules
BOPP	Biaxially Oriented Polypropylene
*ε*_r_	Dielectric Constant
tan *δ*	Dielectric Loss
*U*_d_	Discharge Energy Density
*E*_b_	Breakdown Strength
*T*_g_	Glass Transition Temperature
DFT	Density Functional Theory
MD	Molecular Dynamics
FFV	Fractional Free Volume
NMR	Nuclear Magnetic Resonance
ESI-HRMS	Electron Spray Ionization High Resolution Mass Spectrometry
FT-IR	Fourier Transform Infrared Spectroscopy
XRD	X-Ray Diffraction
MeCN	Acetonitrile
