# Supplementary material for: Supramolecular Engineering of a Homo[2]catenane Filler Enables Polymer Composites with Exceptional High-Temperature Capacitive Energy Storage
Source: Molecules. 2026 May 16;31(10):1691. doi: 10.3390/molecules31101691 (PMC13209518; doi:10.3390/molecules31101691)
Supplement: Supplementary file 1 [file molecules-31-01691-s001.zip › molecules-4289168-supplementary.pdf]

Supporting Information

Supramolecular Engineering of a Homo[2]catenane Filler Enables Polymer Composites with Exceptional High-Temperature Capacitive Energy Storage

Qiao Su[1†], Yan Sun[2†], Jinfeng Li[3], Benteng Ma[1], Xiao Zhang[1], Haifeng Tian[1], Yuheng Ju[1], Saiwen Gao[1], Zhigang, Liu[1], Tian Zhang[3]*, Lin Wu[1]*

[1] High Purity Chemistry Science and Technology Innovation Center of Jilin Province, Centre of Analysis and Measurement, Jilin University of Chemical Technology, 45 Chengde Stress, Jilin, Jilin 132022, China

[2] Key Laboratory of Chemical Waste Resource Utilization of Jilin Province, School of Resources and Environment Engineering, Jilin University of Chemical Technology, 45 Chengde Stress, Jilin, Jilin 132022, China

[3] Electronic Information School, Wuhan University, Wuhan 430072, China

* Correspondence: txz908@whu.edu.cn (T. Z.); wulin@jluct.edu.cn (L.W.)

List of contents of the supplementary information:

Figure S1. Synthesis of **HC^{8+}**.

Figure S2. ^{1}H NMR spectrum (600 MHz, $CDCl_3$, 298K) of compound **1**.

Figure S3. ^{1}H NMR spectrum (600 MHz, $CDCl_3$, 298K) of compound **2**.

Figure S4. ^{1}H NMR spectrum (600 MHz, CD_3CN, 298K) of compound **3**.

Figure S5. ^{1}H NMR spectrum (600 MHz, CD_3CN, 298K) of **DB•2PF_6**.

Figure S6. ^{1}H NMR spectrum (600 MHz, CD_3CN, 298K) of **Blue Box**.

Figure S7. ^{1}H NMR spectrum (600 MHz, CD_3CN, 298K) of **HC8+.**

Figure S8. High-resolution mass spectra (HRMS) of **HC^{8+}**.

Figure S9. Schematic illustration for of the preparation of composition film.

Figure S10. Polymer configuration of (a) PI and (b) PI-HC^{8+}.

Figure S11. SEM images of PI-HC^{8+} composites.

Figure S12. DSC curves of PI and PI-0.5 wt% HC^{8+} composites.

Figure S13. TGA curves of PI and PI-HC^{8+} composites.

Figure S14. Young's modulus of PI and PI-0.5 wt% HC^{8+} composites.

Figure S15. The ε_r and tan δ as a function of frequency for the composites of PI and PI-HC^{8+} composites at room temperature.

Figure S16. The temperature dependence of ε_r and tan δ at 10^3 Hz for PI and PI-0.5 wt% HC^{8+}.

Figure S17. Weibull distribution analysis of the breakdown strength for PI and PI-0.5 wt% HC^{8+} composites at 100 °C.

Figure S18. Weibull distribution analysis of the breakdown strength for PI and PI-0.5 wt% HC^{8+} composites at 150 °C.

Figure S19. TSDC curves of PI and PI-HC^{8+} composites.

Figure S20. Schematic unipolar *D-E* loop of a dielectric material.

Figure S21. *D-E* loops of PI and PI-HC^{8+} composites at 100 °C.

Figure S22. Energy storage performance of pristine PI and PI-HC^{8+} composites at 100 °C.

Figure S23. *D-E* loops of PI and PI-HC^{8+} composites at 150 °C.

Table S1. Batch-to-batch reproducibility of PI-0.5 wt% HC^{8+} composites.

1. Synthetic Details

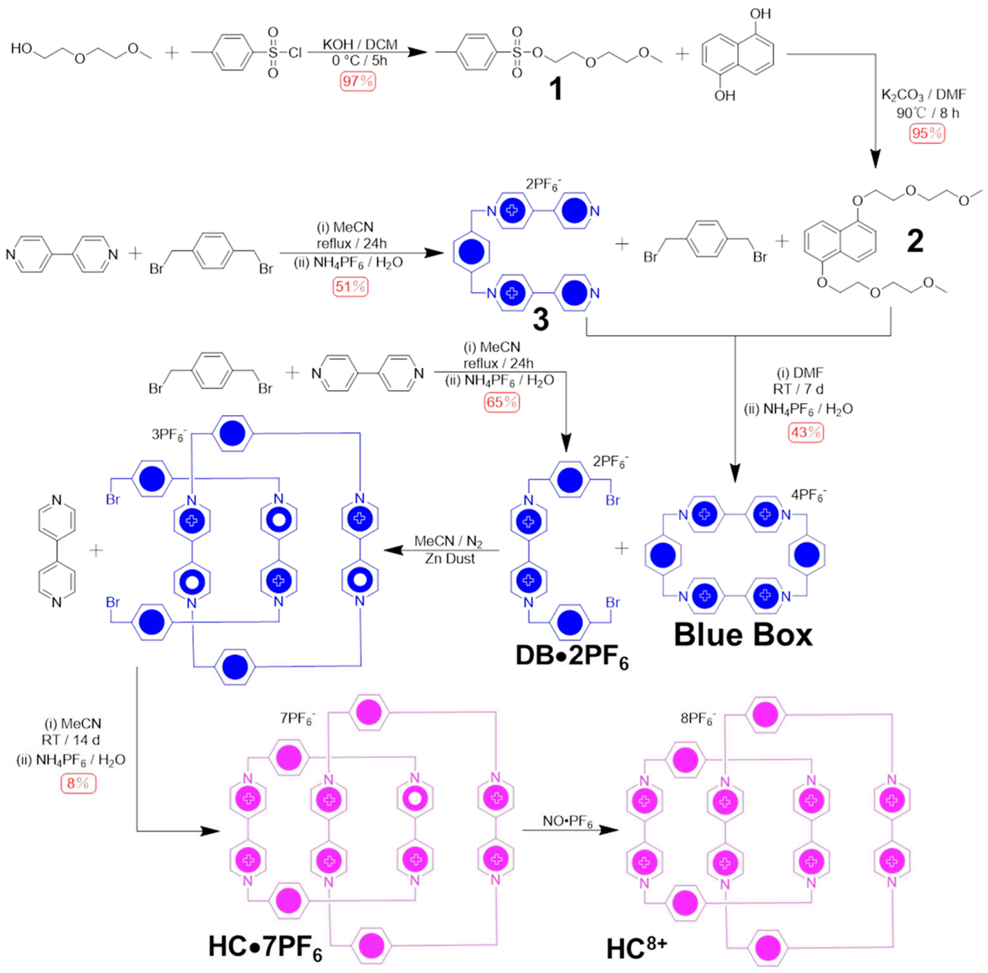

Figure S1. Synthesis of **HC**$^{8+}$.

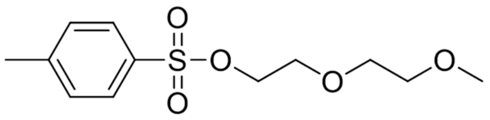

1

1: A mixture of diethylene glycol monomethyl ether (6.72 g, 56.0 mmol, 1.0 equiv), *p*-toluenesulfonyl chloride (10.7 g, 56.0 mmol, 1.0 equiv), and CH_2Cl_2(100 mL) was stirred at in an ice water bath for 10 min. KOH (15.7 g, 280 mmol, 5.0 equiv) was added to this mixture, and it remained in an ice-water bath until the reaction was completed. After quenching the reaction with water, and the mixture was partitioned between CH_2Cl_2 (50 mL) and saturated brine (50 mL). The combined organic phases were dried (Na_2SO_4) and concentrated under vacuum. The residue was purified by flash column chromatography (SiO_2, EtOAc / light petroleum = 1 / 5, v/v) to afford compound **1** as a colorless liquid (14.8 g, 97%). ^{1}H NMR (600 MHz, $CDCl_3$) δ 7.80 (d, J = 8.3 Hz, 2H),

7.34 (d, J = 8.1 Hz, 2H), 4.19 – 4.15 (m, 2H), 3.72 – 3.67 (m, 2H), 3.59 – 3.57 (m, 2H), 3.49 – 3.47 (m, 2H), 3.35 (s, 3H), 2.45 (s, 3H).

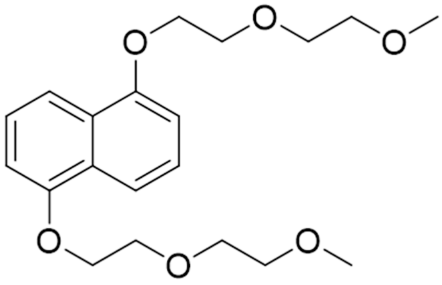

2

2: Compound **1** (3.79 g, 13.8 mmol, 2.1 equiv), 1,5-dihydroxy naphthalene (1.05 g, 6.59 mmol, 1.0 equiv), and K_2CO_3 (3.64 g, 26.4 mmol, 4.0 equiv) were added to a round-bottom flask containing DMF (50 mL) and the reaction mixture was stirred at 90 °C for 8 h. After cooling to room temperature, the reaction mixture was filtered through a pad of Celite and washed with CH_2Cl_2. The combined organic filtrate was washed with brine, dried (Na_2SO_4), and concentrated under vacuum. The residue was purified by flash column chromatography (SiO_2, light petroleum / EtOAc = 5 / 1, v/v) to give **2** as a light green solid (2.27 g, 95%). ^{1}H NMR (600 MHz, $CDCl_3$) δ 7.86 (d, J = 8.4 Hz, 2H), 7.34 (t, J = 8.0 Hz, 2H), 6.84 (d, J = 7.6 Hz, 2H), 4.34 – 4.28 (m, 4H), 4.02 – 3.99 (m, 4H), 3.82 – 3.78 (m, 4H), 3.61 – 3.58 (m, 4H), 3.40 (s, 6H).

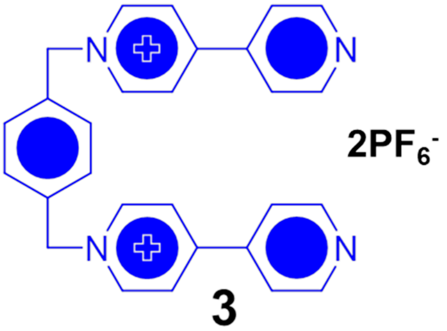

3

3: 4,4'-Bipyridine (5.0 g, 32.1 mmol, 3.5 equiv) and MeCN (100 mL) were stirred at 100 °C for 2 h. Then, 1,4-dibromomethylbenzene (2.42 g, 9.16 mmol, 1.0 equiv) containing MeCN (100 mL) were added dropwise to the boiling mixture, and the reaction mixture was stirred at 100 °C until the reaction was completed. The reaction mixture was filtered and the filter cake was washed with MeCN (50 mL) and Et_2O (50

mL) to give a pale yellow solid. After the solid was dissolved in water (100 mL) and partitioned in CH_2Cl_2 (50 mL). The combined water phases were concentrated under vacuum. The residue was purified by flash column chromatography (SiO_2, MeOH / H_2O / NH_4Cl= 1 / 3 / 1, v/v) and pure fractions were collected, concentrated, added to a saturated NH_4PF_6 solution, and filtered to afford **3** as a white solid (3.31 g, 51%). ^1H NMR (600 MHz, CD_3CN) δ 8.86 – 8.81 (m, 8H), 8.33 (d, J = 6.9 Hz, 4H), 7.79 – 7.76 (dd, J_1 = 4.5 Hz, J_2= 1.7 Hz 4H), 7.55 (s, 4H), 5.77 (s, 4H).

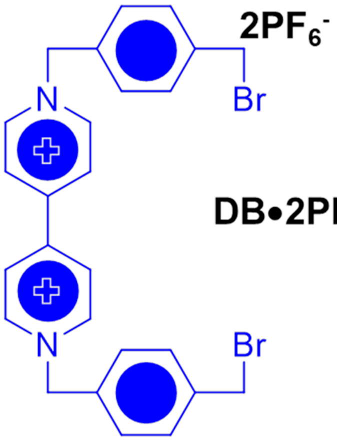

DB•2PF$_6$

DB•2PF6: 1,4-dibromomethylbenzene (2.13 g, 8.07 mmol, 10.0 equiv) and MeCN (25 mL) were stirred at 100 °C for 2 h. Then, 4,4'-Bipyridine (126 mg, 0.81 mmol, 1.0 equiv) containing MeCN (100 mL) were added dropwise to the boiling mixture, and the reaction mixture was stirred at 100 °C until the reaction was completed. The reaction mixture was filtered and the filter cake was washed with MeCN (50 mL) and Et_2O (50 mL) to give a yellow solid. After the solid was dissolved in water (100 mL) and partitioned in CH_2Cl_2 (50 mL). The combined water phases were concentrated under vacuum. The residue was purified by flash column chromatography (SiO_2, MeOH / H_2O / NH_4Cl= 1 / 3 / 1, v/v) and pure fractions were collected, concentrated, added to a saturated NH_4PF_6 solution, and filtered to afford **DB•2PF$_6$** as a white solid (427 mg, 65%). ^1H NMR (600 MHz, CD_3CN) δ 8.95 (d, J = 7.0 Hz, 4H), 8.35 (d, J = 6.4 Hz, 4H), 7.46 (d, J = 2.1 Hz, 8H), 5.79 (s, 4H), 4.61 (s, 4H).

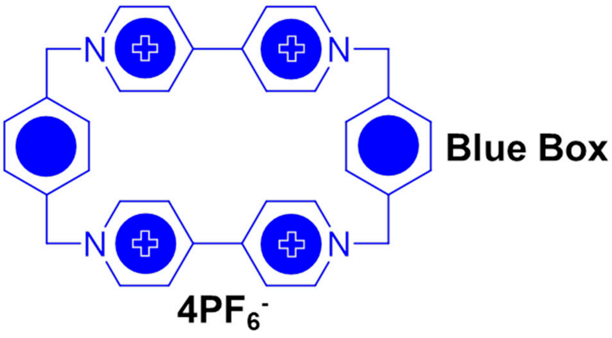

Blue Box : Compound **3** (1.50 g, 1.86 mmol, 1.0 equiv), 1,4-dibromomethylbenzene (623 mg, 2.12 mmol, 1.0 equiv), and Compound **2** (1.54 g, 4.24 mmol, 2.0 equiv) were added to a sealed tube containing DMF (30.0 mL) and the reaction mixture was stirred at room temperature for 5 d. Add ether to precipitate purple solid and the mixture was filtered and the filter cake was washed with CH_2Cl_2 (50 mL) to give purple solid. After the solid was dissolved in water (100 mL) and partitioned in CH_2Cl_2 (50 mL) until the water phase is colorless. The combined water phases were concentrated under vacuum. The residue was purified by flash column chromatography (SiO_2, MeOH / H_2O / NH_4Cl= 1 / 3 / 1, v/v) and pure fractions were collected, concentrated, added to a saturated NH_4PF_6 solution, and filtered to afford **3** as a white solid (1.12 g, 43%). ^1H NMR (600 MHz, CD_3CN) δ 8.87 (d, J = 7.0 Hz, 8H), 8.17 (d, J = 7.0 Hz, 8H), 7.53 (s, 8H), 5.75 (s, 8H).

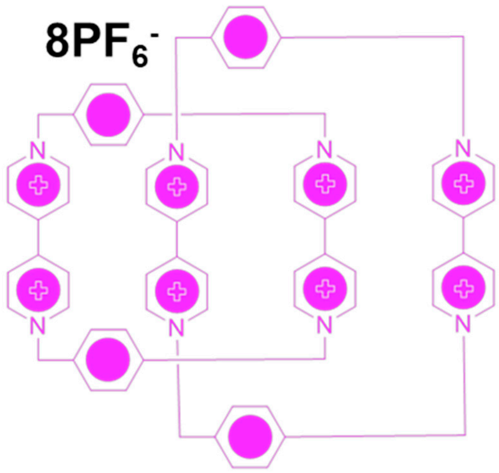

HC^8+^

HC^{8+}: Blue Box (647 mg, 0.60 mmol, 1.0 equiv) and DB•2PF6 (573 mg, 0.70 mmol, 1.2 equiv) were dissolved in degassed MeCN (35 mL) in a 100-mL round-bottomed

flask in a glovebox. An excess of Zn dust (~57 mg) was added under vigorous stirring to this solution. After 30 min, the solution turned from colorless to a deep purple color. The excess of Zn dust was filtered off. The purple filtrate was collected in another round-bottomed flask and 4,4'-bipyridine (110 mg, 0.70 mmol, 1.2 equiv) was added to it. The resulting mixture was allowed to stand for 2 weeks at room temperature before being removed from the glovebox and the solvent evaporated off under vacuum. The resulting deep purple solid was purified by flash column chromatography (SiO_2, MeOH / H_2O / NH_4Cl = 1 / 3 / 1, v/v). Pure fractions were collected, concentrated, added to a saturated NH_4PF_6 solution, and filtered to afford HC•7PF_6 as a purple solid (21 mg, 8%). HC•7PF_6 (97 mg) was oxidized to **HC^{8+}** (45 mg) by the addition of an excess of NO•PF_6. ^{1}H NMR (600 MHz, CD_3CN): δ 8.99 (d, J = 6.4 Hz, 8H), 8.84 (d, J = 6.4 Hz, 8H), 8.24 (d, J = 8.2 Hz, 8H), 8.11 (d, J = 8.2 Hz, 8H), 7.73 (d, J = 6.4 Hz, 8H), 6.10 (s, 8H), 5.97 (s, 8H), 4.22 (d, J = 6.4 Hz, 8H). ESI-HRMS for **HC^{8+}**, Calcd for $C_{72}H_{64}F_{48}N_8P_8$: m/z = 1910.3104 [M – 2PF_6]$^{2+}$, 1765.3462 [M – 3PF_6]$^{3+}$; found: 1910.3000 [M – 2PF_6]$^{2+}$, 1765.3363 [M – 3PF_6]$^{3+}$.

2. NMR Spectroscopy

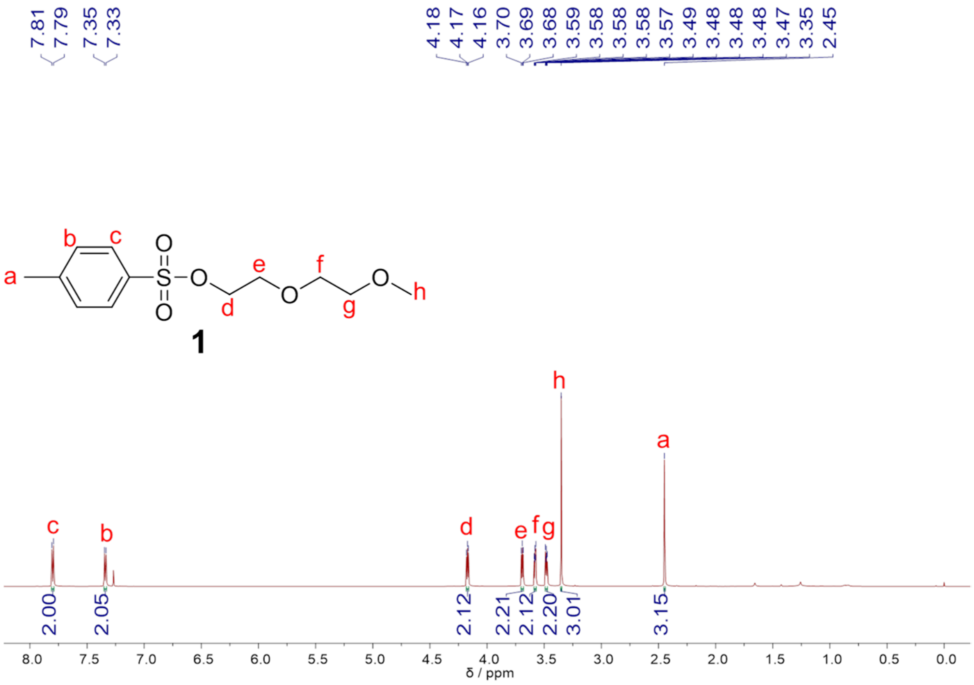

Figure S2. ^{1}H NMR spectrum (600 MHz, $CDCl_3$, 298K) of compound **1**.

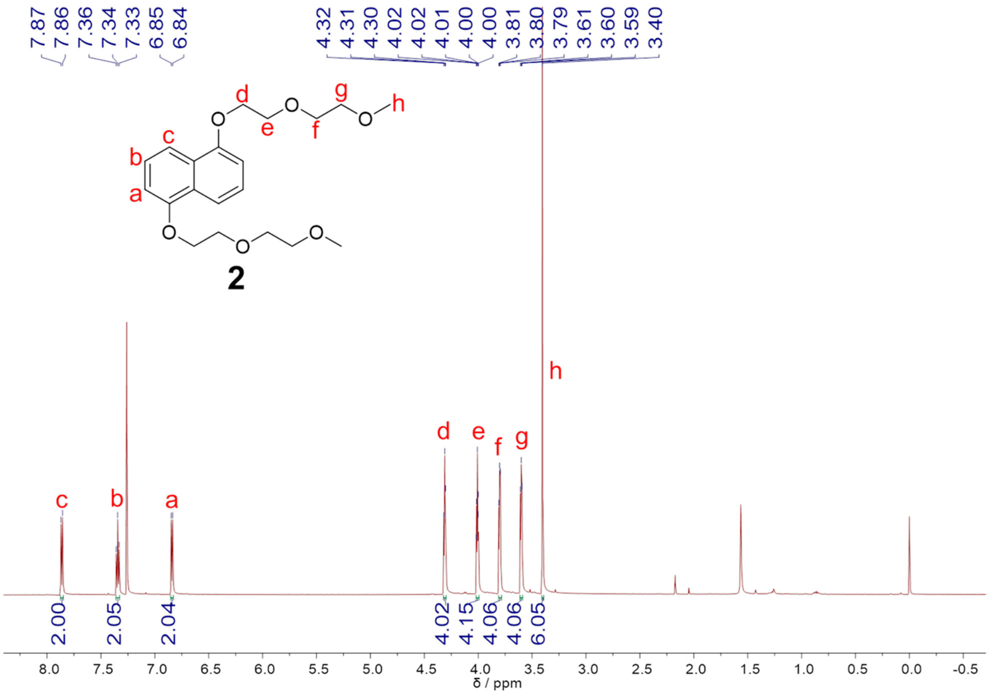

Figure S3. ^{1}H NMR spectrum (600 MHz, $CDCl_3$, 298K) of compound **2**.

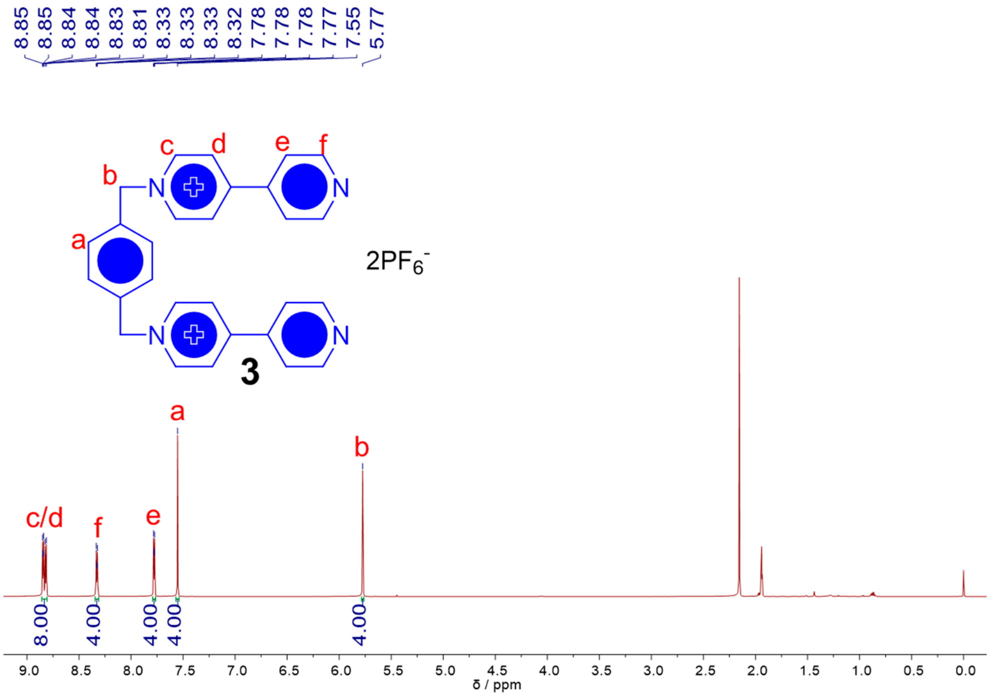

Figure S4. ^{1}H NMR spectrum (600 MHz, CD_3CN, 298K) of compound **3**.

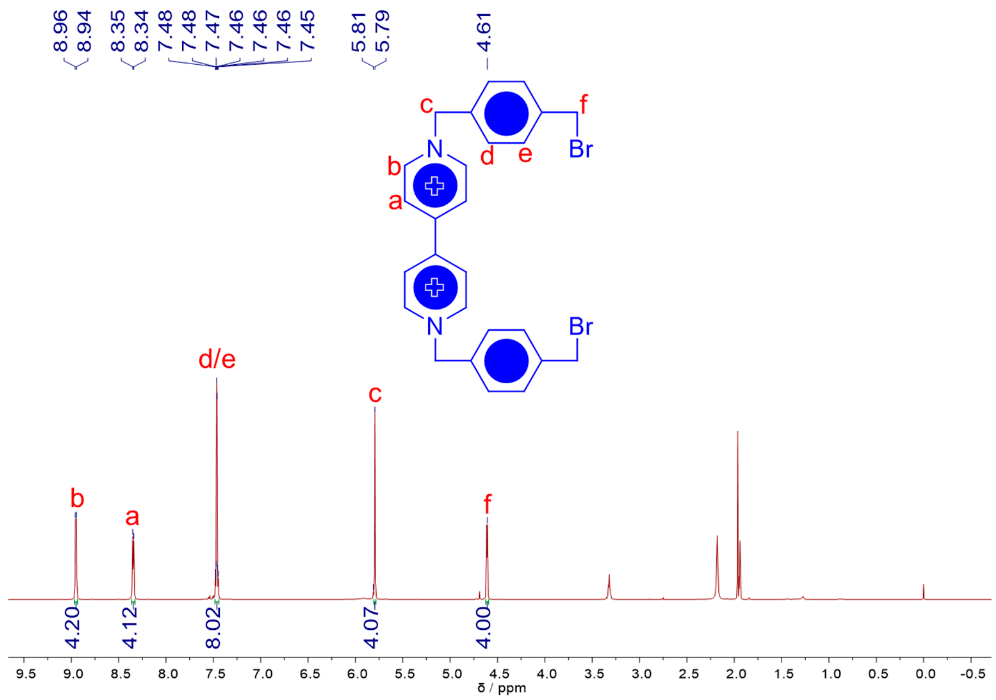

Figure S5. ^{1}H NMR spectrum (600 MHz, CD_3CN, 298K) of **DB•2PF$_6$**.

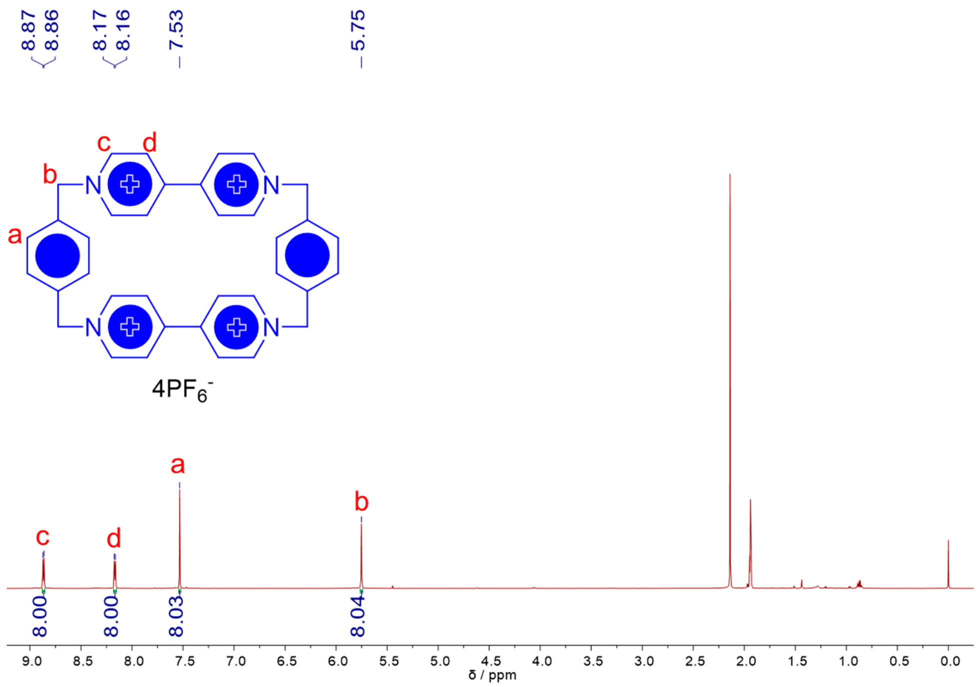

Figure S6. ^{1}H NMR spectrum (600 MHz, CD_3CN, 298K) of **Blue Box**.

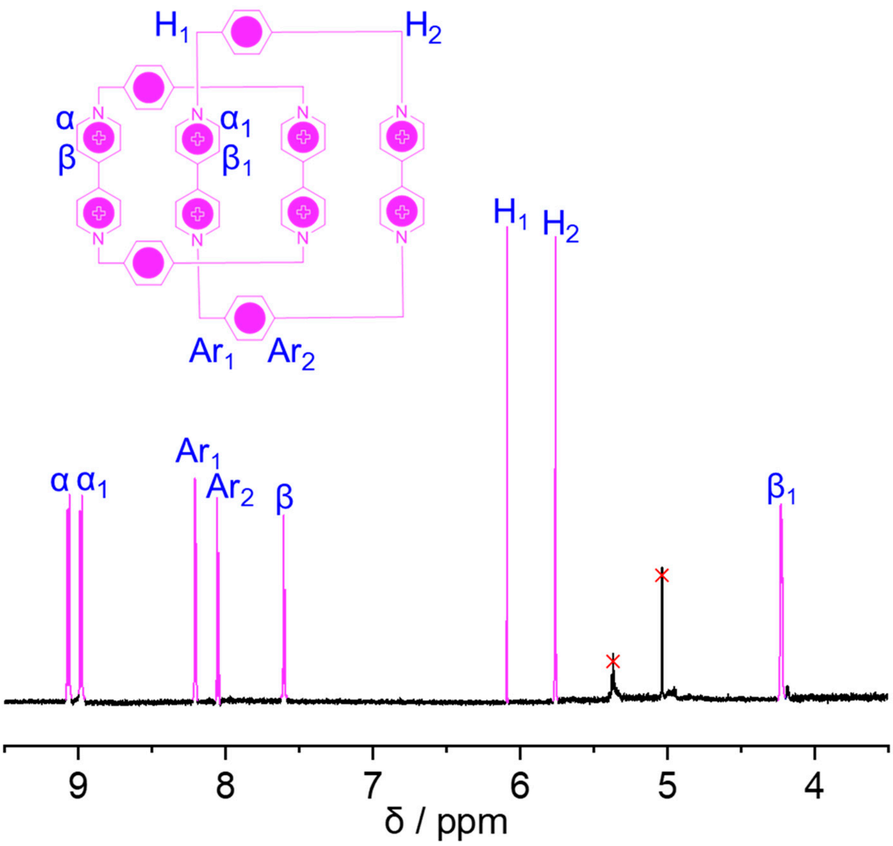

Figure S7. ^{1}H NMR spectrum (600 MHz, CD_3CN, 298K) of **HC8+.**

3. High-Resolution Mass Spectrometry

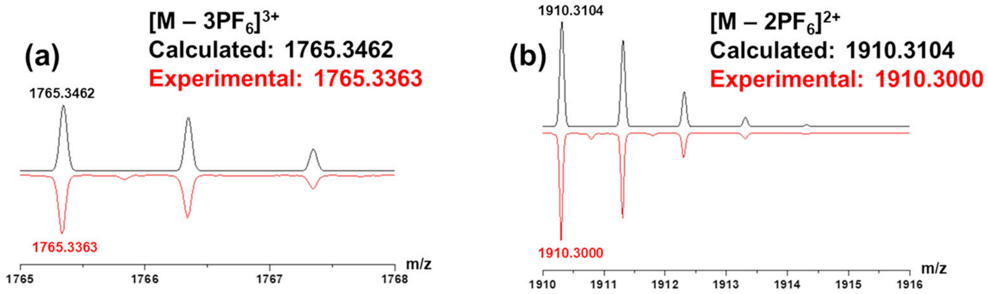

Figure S8. High-resolution mass spectra (HRMS) of $\mathbf{HC^{8+}}$. The experimentally obtained isotopic distribution patterns (red) for (a) ($[M-3PF_6]^{3+}$) and (a) ($[M - 2PF_6]^{2+}$) correlate well with the calculated patterns (black), respectively.

4. Film Preparation

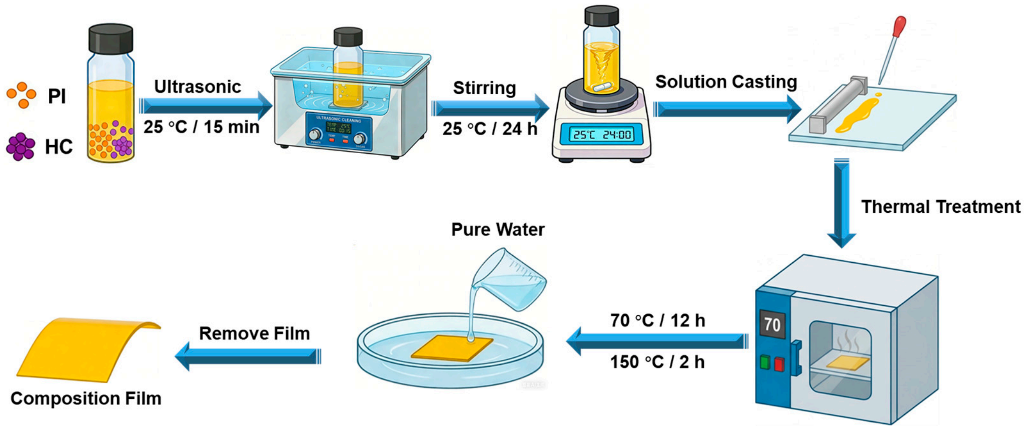

Figure S9. Schematic illustration for of the preparation of composition film.

5. Results and Discussion

5.1 Functional Theory (DFT) Calculation

All calculations were carried out by Gaussian 09 W and GaussView 6.0. The geometry optimization of PI and PI-HC8+ was performed at the B3LYP/6-31G(d) level. The visualization of electrostatic potential distribution was characterized by GaussView 6.0. And the density of state (DOS) was visualized by Multiwfn (Version 3.8).

5.2 Molecular Dynamics (MD) Simulation

Different models with varying proportions were developed using the AC module of the MS software in the MD simulations. To achieve stable configurations, the constructed models underwent geometric structure optimization. Iterative optimization was employed to determine the optimal force field parameters, using Compass as the selected force field, combined with the force field assigned charge distribution method and the smart optimization calculation method. The microcanonical ensemble (NVT) was chosen for the MD calculations, utilizing the Nose thermostat for temperature control and the Velocity Verlet algorithm applied to resolve Newton's equations of motion. The van der Waals interactions were addressed using an atom-based method with a cutoff radius set at 12.5 Å, and the simulation was conducted for 500 ps with a time step of 1 fs. The resulting data were imported into the MS software to determine the appropriate polymer configurations for calculating the free volume, occupied volume, and binding energy.

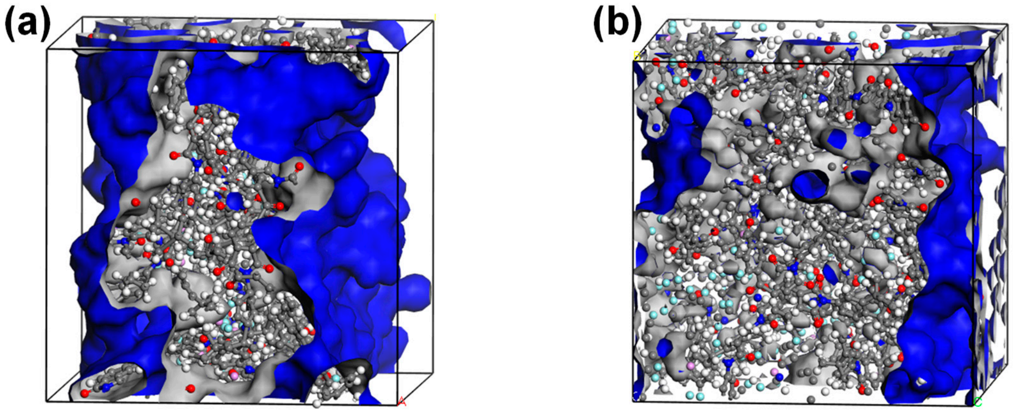

Figure S10. Polymer configuration of (a) PI and (b) PI-HC^{8+}.

5.3 Finite Element Method Simulations

To visualize the temperature distribution within dielectric-film capacitors under elevated-temperature operation, finite-element simulations were carried out in COMSOL Multiphysics. Heat transport in both PI and PI-HC^{8+} films was described by Equation (1):

$$\rho C_p \frac{\partial T}{\partial t} = K\nabla^2 T + Q$$

where ρ, C_p, t, T, K and Q denote the density, specific heat capacity, time, temperature, thermal conductivity, and volumetric heat-generation rate, respectively. The heat-generation term Q was estimated from the dielectric energy dissipation extracted from the *D*-*E* loops according to $Q = f \times U_{loss}$, where U_{loss} is the energy loss measured at 300 MV / m and f is the operating frequency. The simulated geometry was a cylinder with a radius of 5 mm and a height of 10 mm, and the ambient temperature was set to 150 °C.

5.4 Statistical Analysis

To determine the breakdown strength (E_b) of dielectrics. At least 15 points were selected from different regions of each samples and the E_b of each point was tested. The two-parameter Weibull statistic was used to analyze the results as:

$$P(E) = 1 - \exp[-(E/E_b)\,\beta\,]$$

where P(E) is the cumulative probability of electric failure, E is the measured breakdown field of each point, E_b is the electric field for which there is a 63%

probability for the sample to breakdown (Weibull breakdown strength), the shape parameter β evaluates the scatter of data.

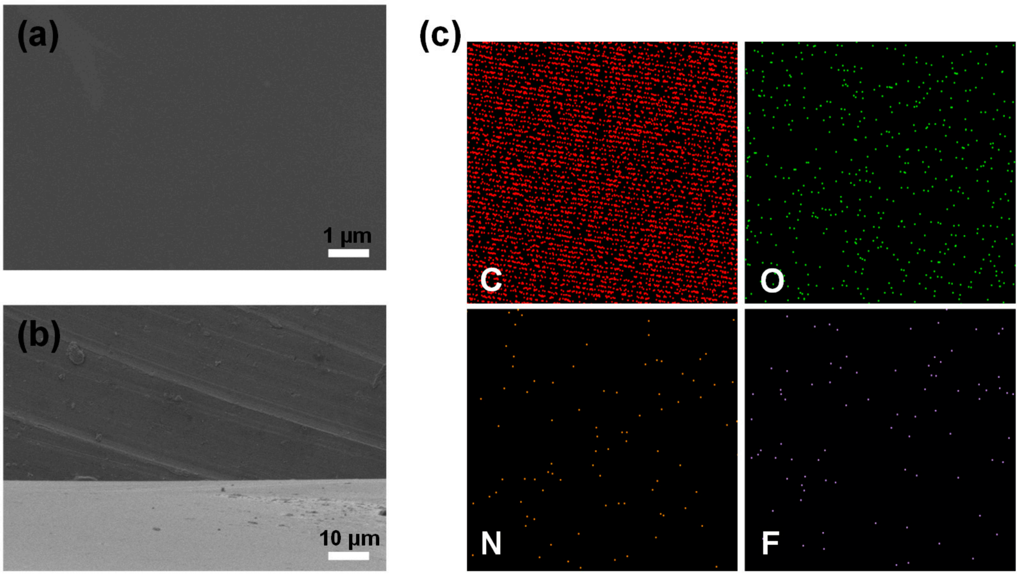

Figure S11. (a) SEM images of PI-HC^{8+} composites, accompanied by corresponding elemental mapping image (c) for C, O, N, and F elements. (b) Cross-sectional SEM image of raw PI.

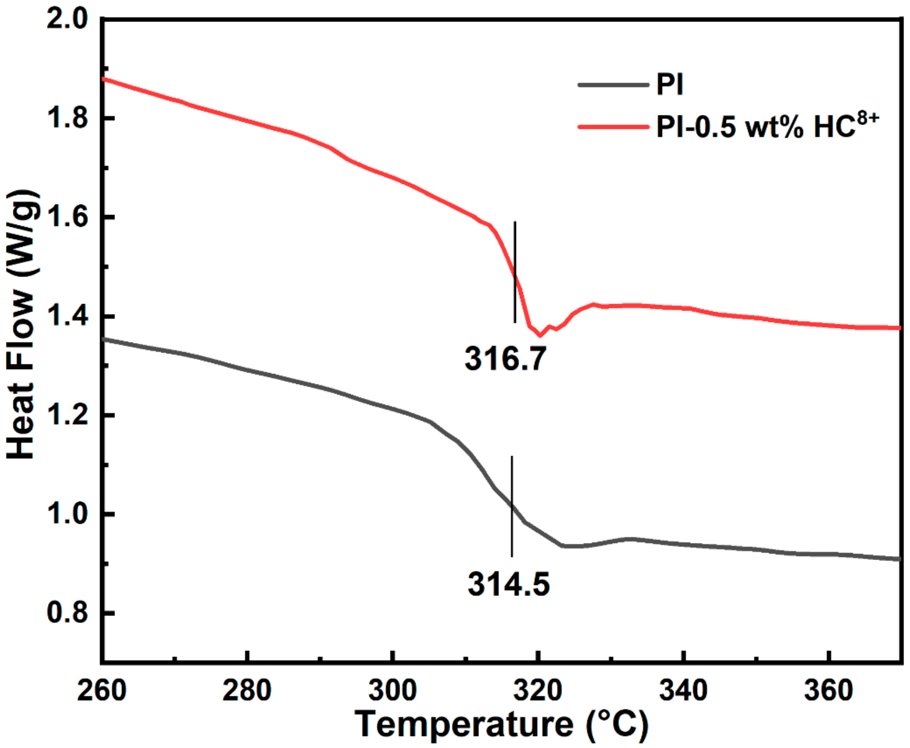

Figure S12. DSC curves of PI and PI-0.5 wt% HC^{8+} composites.

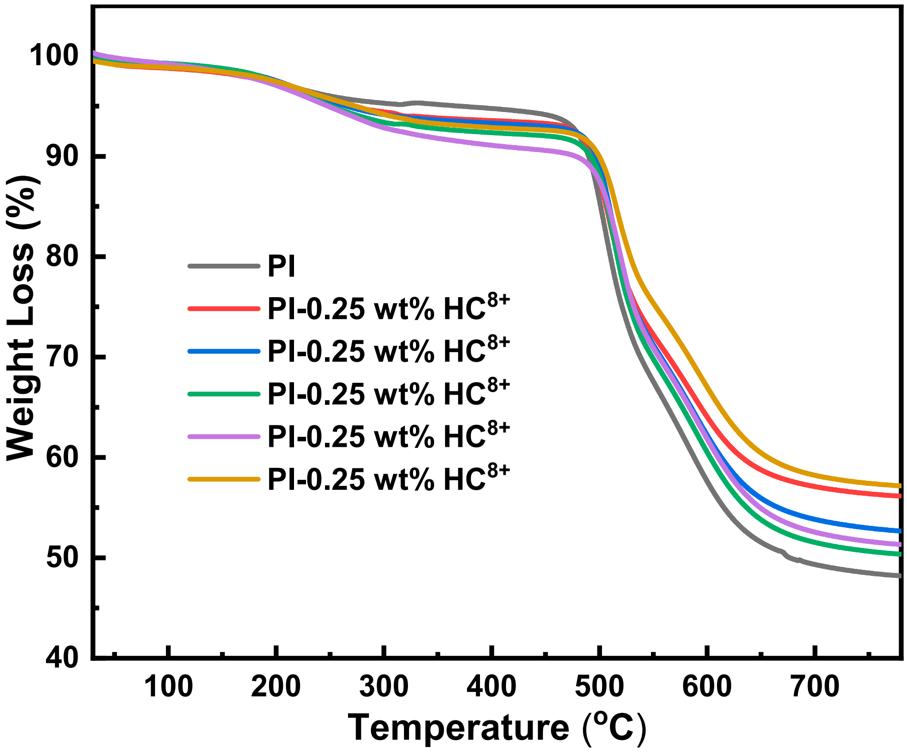

Figure S13. TGA curves of PI and PI-HC^{8+} composites.

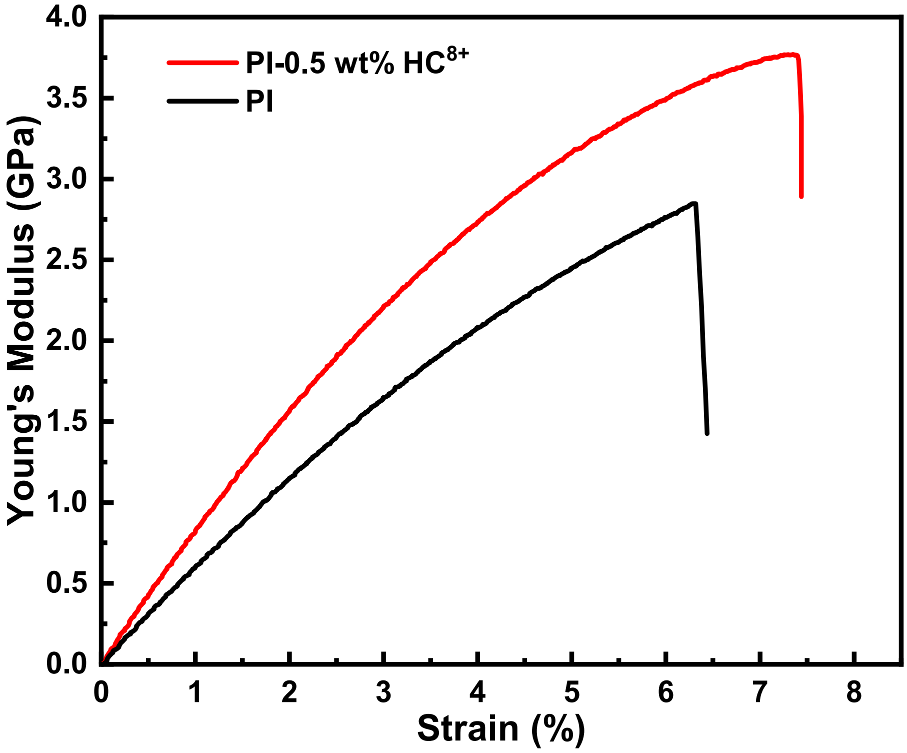

Figure S14. Young's modulus of PI and PI-0.5 wt% HC^{8+} composites.

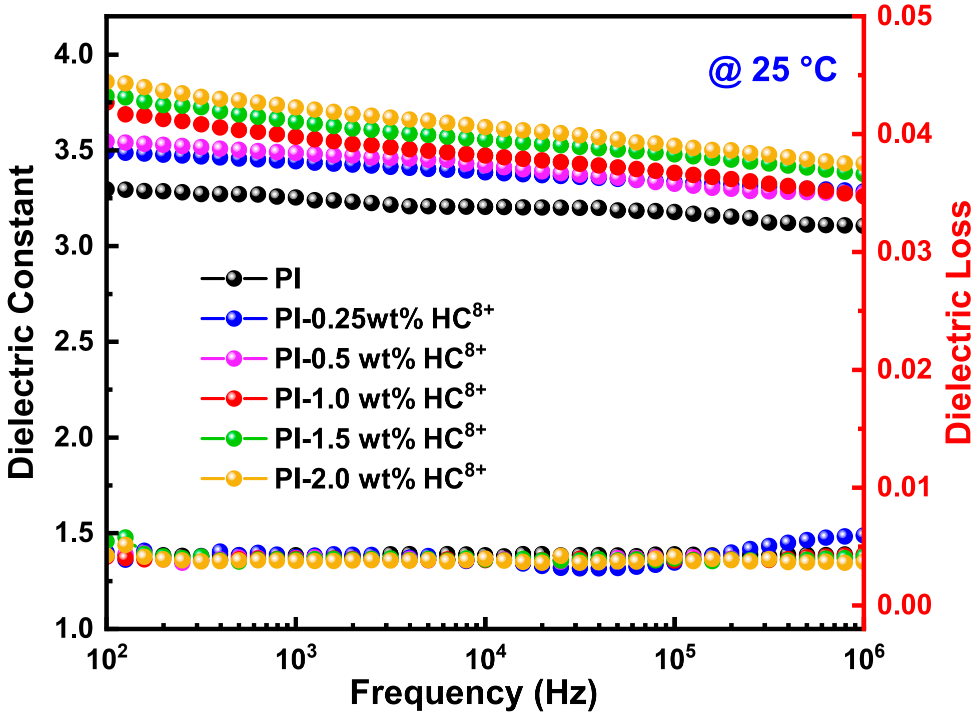

Figure S15. The ε_r and tan δ as a function of frequency for the composites of PI and PI-HC^{8+} composites at room temperature.

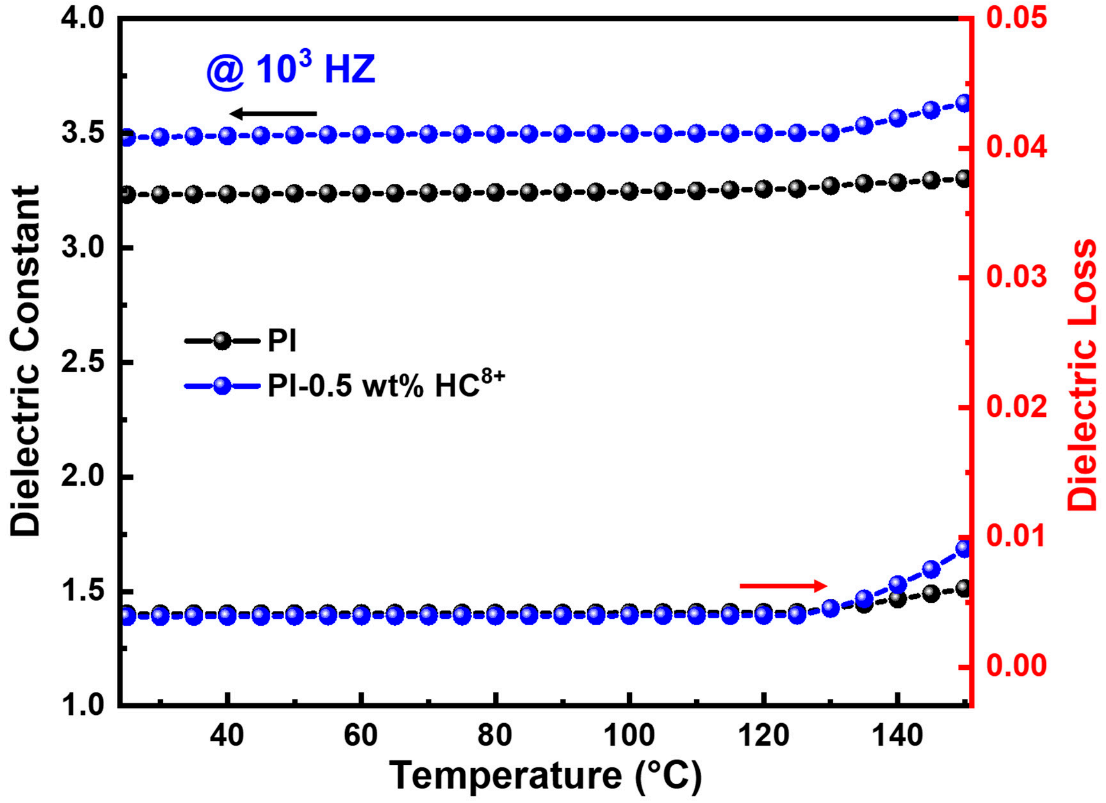

Figure S16. The temperature dependence of ε_r and tan δ at 10^3 Hz for PI and PI-0.5 wt% HC^{8+}.

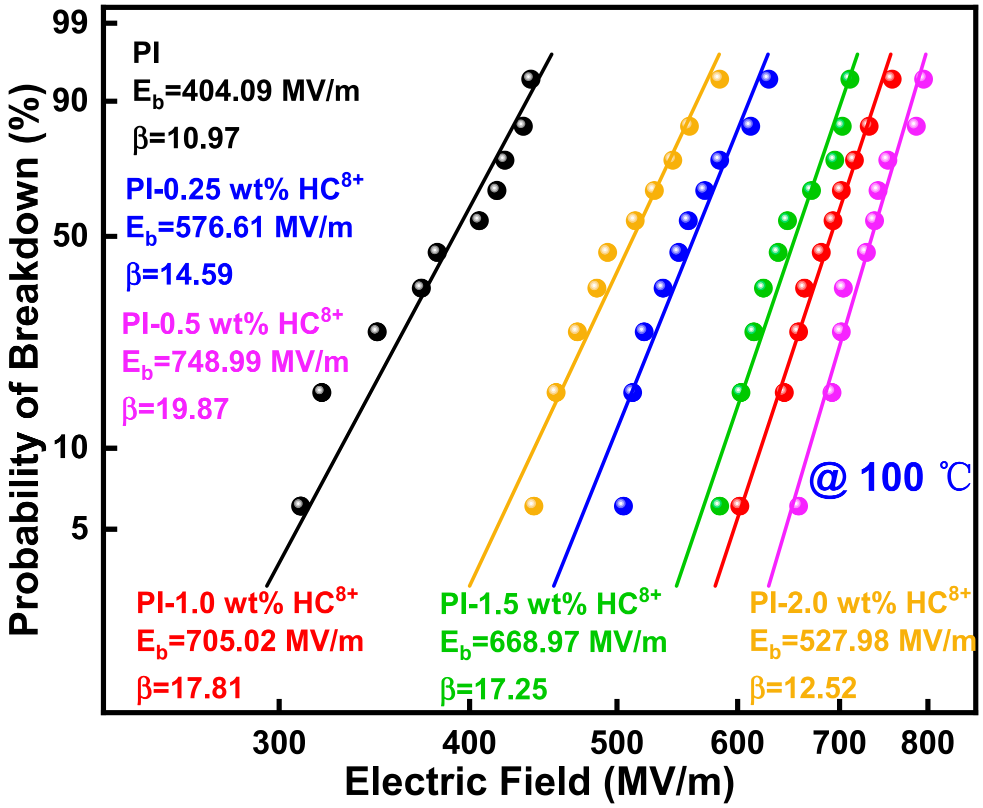

Figure S17. Weibull distribution analysis of the breakdown strength for PI and PI-0.5 wt% HC^{8+} composites at 100 °C.

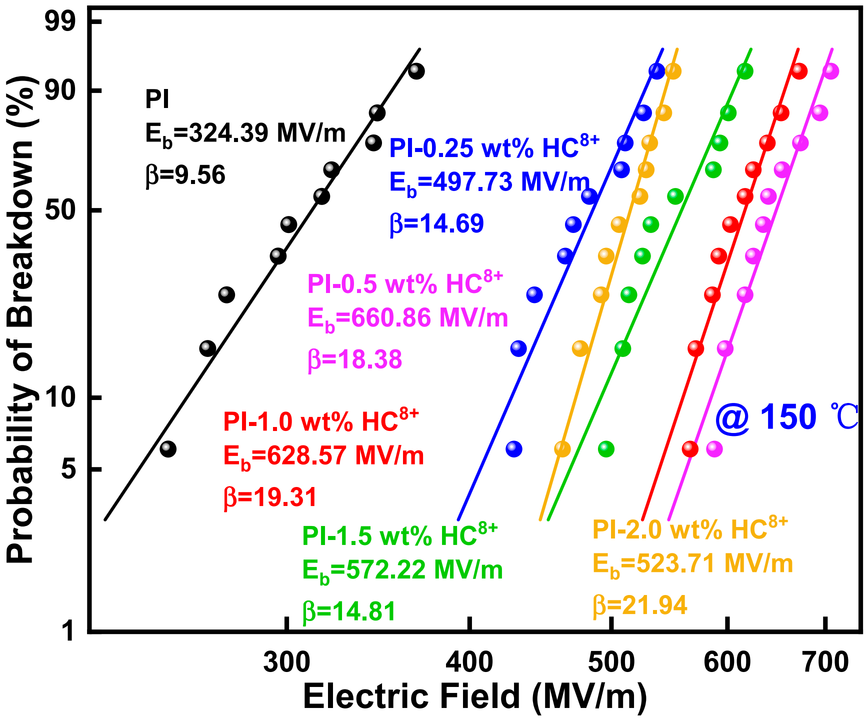

Figure S18. Weibull distribution analysis of the breakdown strength for PI and PI-0.5 wt% HC^{8+} composites at 150 °C.

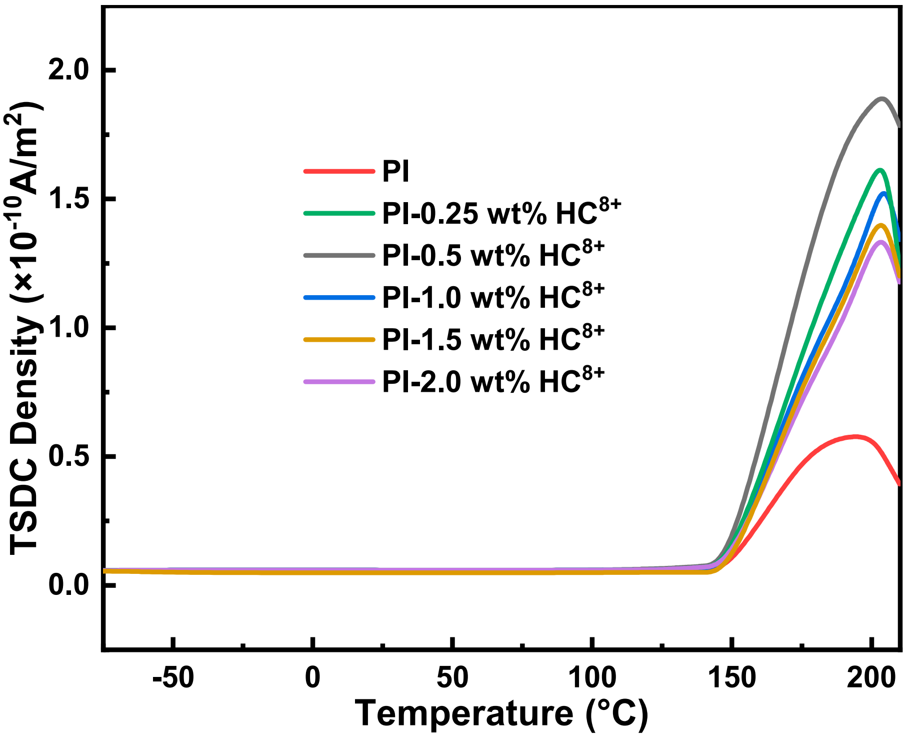

Figure S19. TSDC curves of PI and PI-HC^{8+} composites.

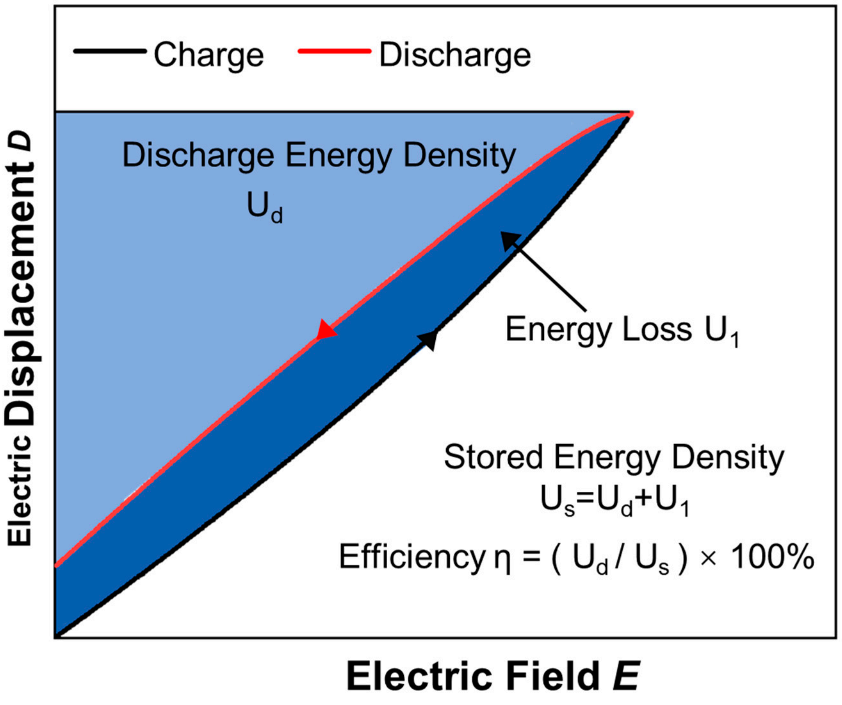

Figure S20. Schematic unipolar *D-E* loop of a dielectric material. The discharged energy density (U_d) is represented by the area (colored in light blue) bounded by the line of discharge, the D axis and the horizontal line. The energy loss (U_l) is represented by the area (colored in dark blue) bounded by lines of charge and discharge, and the D axis. The stored energy density $U_s = U_d + U_l$. The discharge efficiency can be calculated by using the equation $\eta = (U_d / U_s) \times 100\%$.

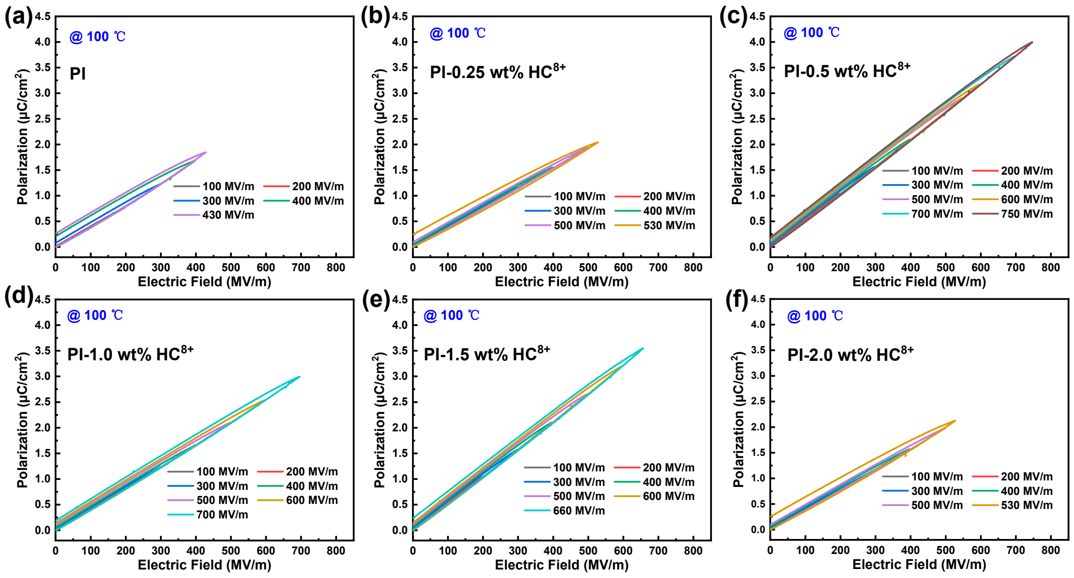

Figure S21. *D-E* loops of PI and PI-HC^{8+} composites at 100 °C.

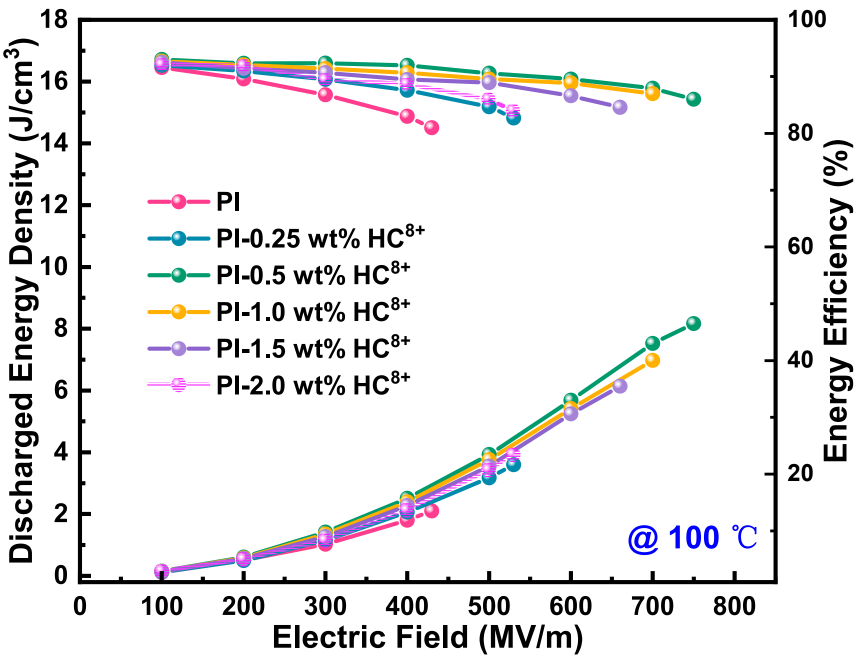

Figure S22. Energy storage performance of pristine PI and PI-HC^{8+} composites at 100 °C.

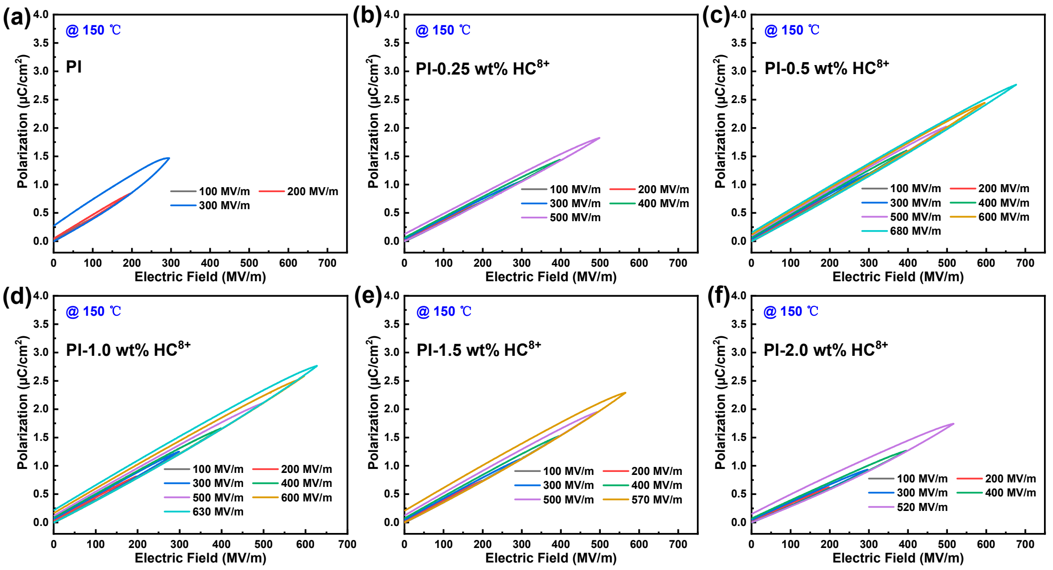

Figure S23. *D-E* loops of PI and PI-HC^{8+} composites at 150 °C.

Batch No.	U_d (J/cm^3) at 600 MV/m, 150 °C	E_b (600 MV/m) at 150 °C
1	6.78	660.8
2	6.81	663.4
3	6.85	655.9
4	6.83	658.8
5	6.79	657.3
Mean ± SD	6.81 ± 0.03	659.2 ± 2.9
CV (%)	0.42%	0.45%

Table S1. Batch-to-batch reproducibility of PI-0.5 wt% HC^{8+} composites.
